# A Single-Molecule View at Nanoparticle Targeting Selectivity: Correlating Ligand Functionality and Cell Receptor Density

**DOI:** 10.1021/acsnano.1c08277

**Published:** 2022-03-11

**Authors:** Laura Woythe, Pranav Madhikar, Natalia Feiner-Gracia, Cornelis Storm, Lorenzo Albertazzi

**Affiliations:** † Department of Biomedical Engineering, Institute for Complex Molecular Systems (ICMS), Eindhoven University of Technology, Eindhoven 5612AZ, The Netherlands; ‡ Institute for Bioengineering of Catalonia (IBEC), The Barcelona Institute of Science and Technology (BIST), Barcelona 08036, Spain; § Department of Applied Physics, Institute for Complex Molecular Systems (ICMS), Eindhoven University of Technology, Den Dolech 2, 5600MB Eindhoven, The Netherlands

**Keywords:** nanomedicine, active targeting, dSTORM, heterogeneity, nanoparticle functionality, super-resolution microscopy

## Abstract

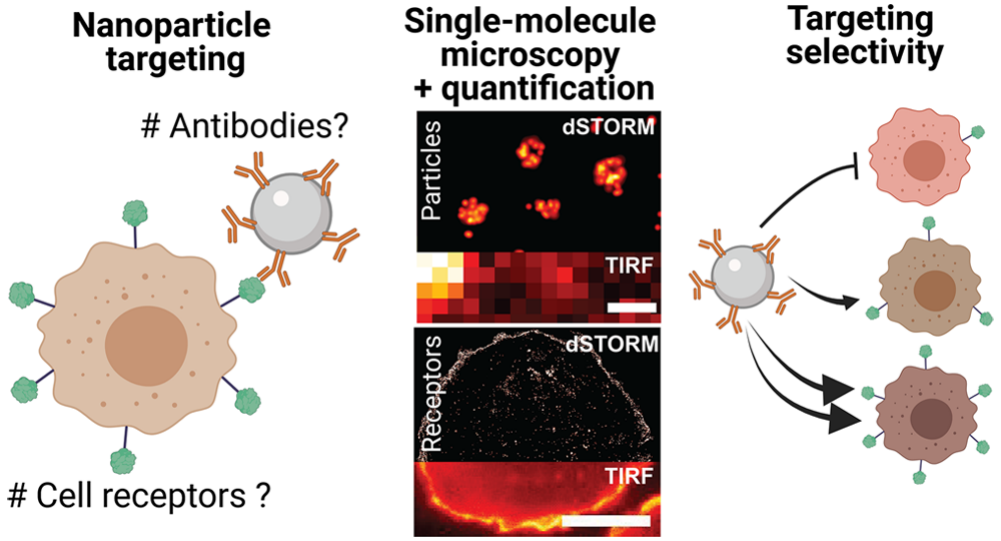

Antibody-functionalized nanoparticles (NPs) are commonly used to increase the targeting selectivity toward cells of interest. At a molecular level, the number of functional antibodies on the NP surface and the density of receptors on the target cell determine the targeting interaction. To rationally develop selective NPs, the single-molecule quantitation of both parameters is highly desirable. However, techniques able to count molecules with a nanometric resolution are scarce. Here, we developed a labeling approach to quantify the number of functional cetuximabs conjugated to NPs and the expression of epidermal growth factor receptors (EGFRs) in breast cancer cells using direct stochastic optical reconstruction microscopy (dSTORM). The single-molecule resolution of dSTORM allows quantifying molecules at the nanoscale, giving a detailed insight into the distributions of individual NP ligands and cell receptors. Additionally, we predicted the fraction of accessible antibody-conjugated NPs using a geometrical model, showing that the total number exceeds the accessible number of antibodies. Finally, we correlated the NP functionality, cell receptor density, and NP uptake to identify the highest cell uptake selectivity regimes. We conclude that single-molecule functionality mapping using dSTORM provides a molecular understanding of NP targeting, aiding the rational design of selective nanomedicines.

Active targeting of NPs is widely used to improve their selectivity toward the cell or tissue of interest. For example, antibodies are commonly conjugated to NPs to promote selective targeting.^1,2^ However, the clinical translation of active-targeted NPs is challenging, with no approved formulation so far.^3^ The limited clinical success indicates that a better understanding of the pitfalls encountered in active-targeting of NPs is needed.

The number of NP ligands and the target cell receptor density are two critical parameters to be considered to maximize the performance of active targeting.^4^ The characterization of these parameters is challenging due to their nanometric size and their often low abundance, making them hardly detectable by standard characterization techniques.^5,6^ Consequently, these numbers are rarely reported in the literature and are based on assumptions rather than measured quantities. Several ensemble approaches have been developed to characterize cell receptors and NP functionality, including fluorescence-activated cell sorting^7,8^ and fluorescence/radiolabeling.^9,10^ However, ensemble techniques are often indirect measurements and are limited by their lack of information at the single-molecule level, disregarding structural heterogeneities.^4^ Targeting of cells occurs at a molecular level; thus, direct methods that count the specific number of ligands and receptors with single-molecule resolution are highly desired to understand their interaction.^11,12^

Single-molecule microscopy techniques provide the necessary resolution and sensitivity to quantify the distribution of molecules and the underlying heterogeneity of nanostructures at a single-receptor or single-NP level.^13−15^ Furthermore, these microscopy techniques can be expanded to quantify the functionality of molecules. For example, transmission electron microscopy (TEM) and single-molecule localization microscopy (SMLM) have been used to quantify the number and map the position of functional sites of proteins on the surface of single NPs.^16−18^ Unlike ensemble techniques, single-molecule characterization techniques allow the quantification of functional heterogeneity in nanostructures. This feature is crucial when considering the NP–cell interaction, as nonfunctional or unfavorable orientations of NP ligands might bring adverse effects such as off-target accumulation in the liver.^19^

In this work, we developed a functional single-molecule labeling approach to quantify the functionality of cetuximab-conjugated silica NPs and the density of EGFRs on breast cancer cell lines using direct stochastic optical reconstruction microscopy (dSTORM). dSTORM is an SMLM technique with excellent resolution (around 20 nm) that allows distinguishing individual cell receptors and ligands by quantifying single fluorescent events.^20,21^ This feature provides single-molecule statistics on the distribution and heterogeneity of these nanostructures.^22,23^ The functional labeling approach is inspired by the targeting interaction, consisting of (1) a recombinant EGFR probe to map the functional cetuximab conjugated to NPs and (2) cetuximab to label accessible EGFR expressed in cells. The single-molecule mapping of functional ligands (cetuximab) on NPs and receptors (EGFR) on cells allows the molecular understanding behind the NP targeting selectivity observed in NP uptake experiments.

Combined with a geometrical model, we found that only a minor fraction of conjugated antibodies are accessible for targeting after a nonoriented coupling method. Furthermore, the functionality of antibodies does not scale linearly with the total number of antibodies conjugated to NPs above a certain threshold. Besides NP functionality, the density of EGFR receptors was quantified using dSTORM in distinct breast cancer cell lines, revealing inter- and intracellular heterogeneities.

We note that it is crucial to consider a threshold of both parameters, NP functionality and receptor density, in the design of NPs that target a specific cell population with high selectivity (i.e., receptor overexpressing cancer cells). Here, dSTORM proves to be a powerful technique for quantifying NP ligands and cell receptor densities at a single-molecule level and thus a valuable tool for the rational design of active-targeted drug delivery.

## Results and Discussion

**Figure 1:**
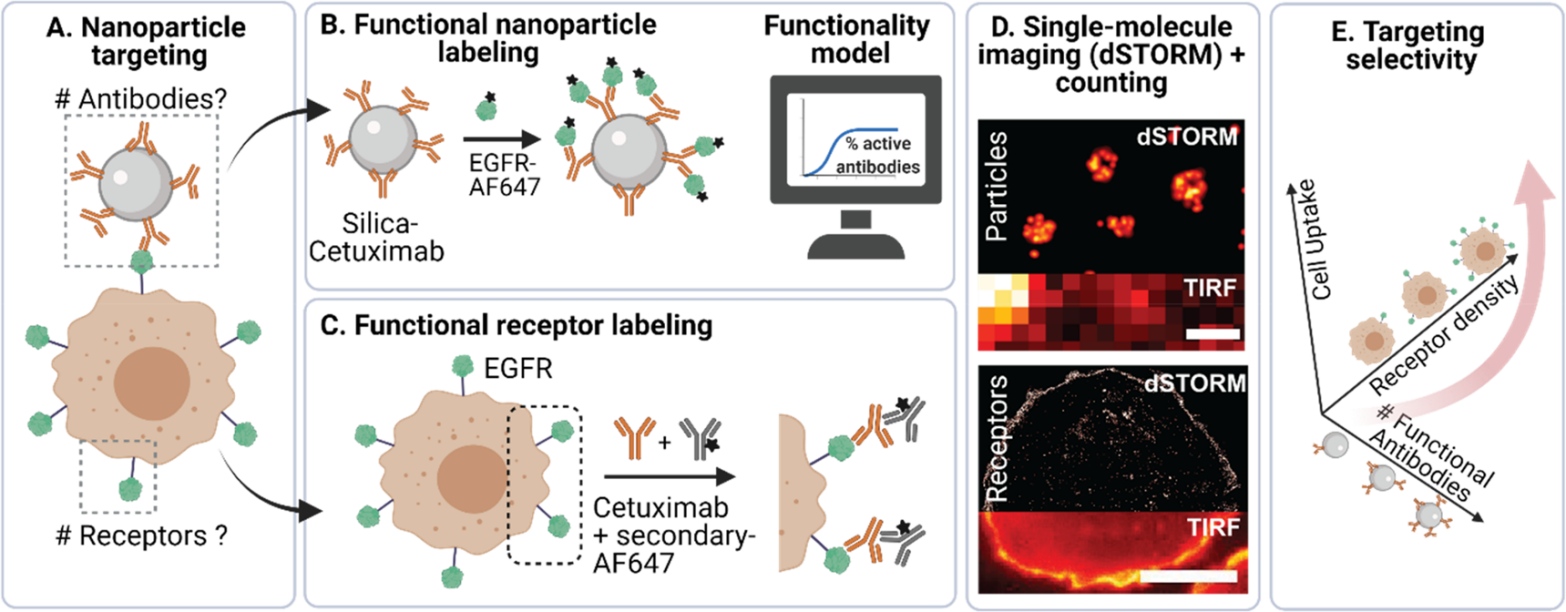
Schematic representation of dSTORM imaging and quantification of functional silica-cetuximab NPs and cell surface EGFR. (A) To understand NP targeting efficiency, information about the functional number of antibodies on NPs and the number of EGFR receptors on the target cell is required. (B) NP functionality was mapped using a labeled EGFR probe. A geometrical model was developed to calculate the expected functionality of antibody-conjugated NPs. (C) EGFR on breast cancer cells was mapped using cetuximab and a labeled secondary antibody, thus revealing potential silica-cetuximab binding sites. (D) dSTORM was used to image and quantify labeled NPs and EGFR on cells at a single-molecule level. A conventional TIRF image is shown for comparison. Scale bar particles 400 nm and receptors 10 μm. (F) The information on both parameters was combined to understand the targeting selectivity of silica-cetuximab NPs toward cells with different EGFR expressions.

To understand NP targeting efficiency at the molecular level, we developed a single-molecule method to characterize the number of functional NP ligands and the receptor density on the target cell using dSTORM (Figure 1). We implemented a functional labeling approach based on the interaction between the therapeutic antibody cetuximab and the EGFR. On the one hand, functional cetuximabs conjugated to silica NPs were detected using a fluorescent recombinant EGFR probe, consisting of the receptor’s extracellular domain. Thus, only cetuximabs with accessible and intact fragment antigen-binding (Fab) regions were imaged (Figure 1B). On the other hand, EGFR on the cell surface is detected with cetuximab, thus elucidating all the possible NP targeting sites (Figure 1C). In contrast to qualitative methods, dSTORM allows counting at a single-molecule level of both antibodies and receptors (Figure 1D). This single-molecule quantification can be used to elucidate the effects of varying the number of functional antibodies on the NP and the cell receptor density on NP cell uptake (Figure 1E).

### dSTORM Imaging of Silica-Cetuximab NPs Allows Antibody Quantification

**Figure 2:**
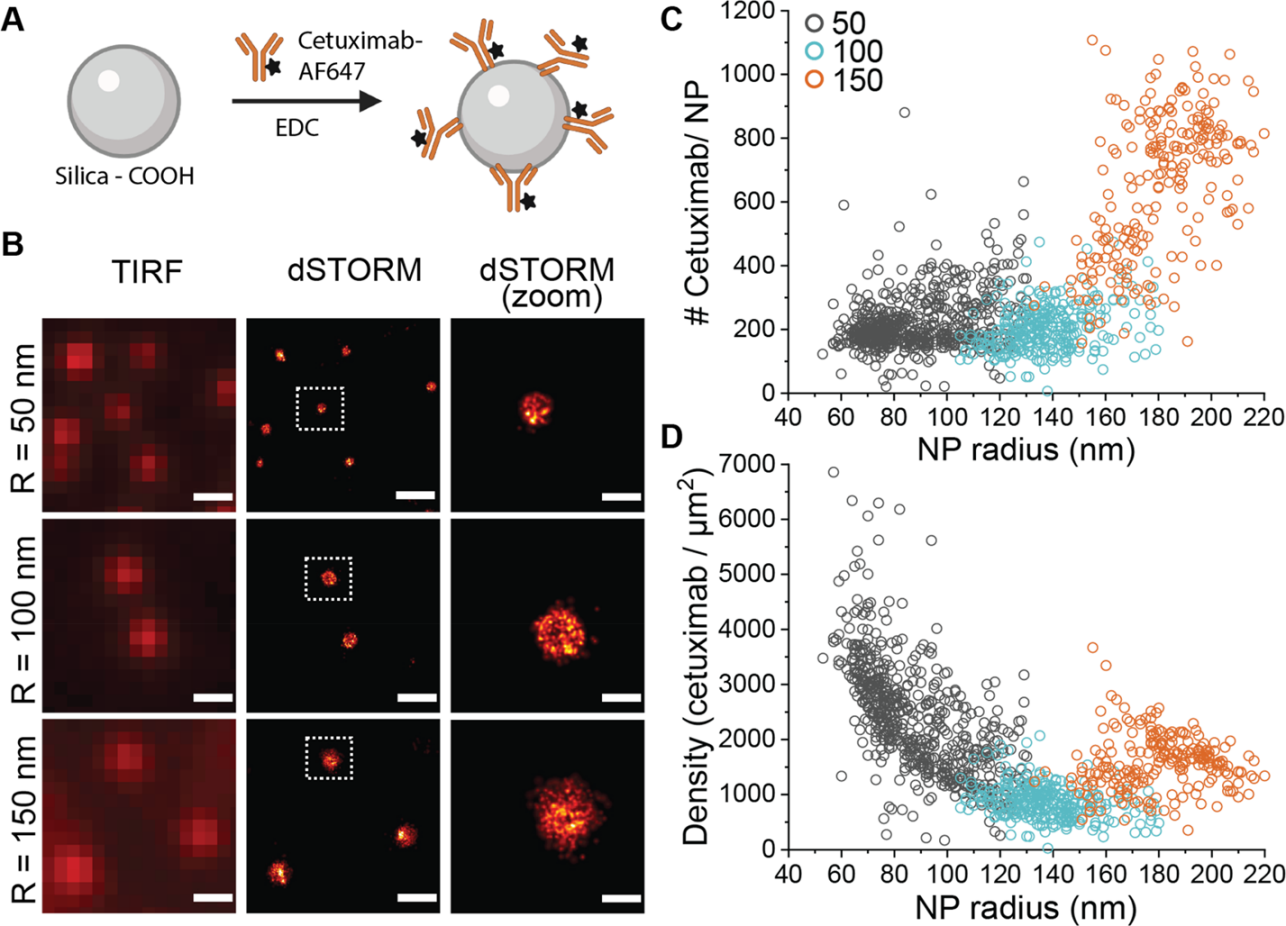
dSTORM imaging of cetuximab-AF647-conjugated silica NPs. (A) Schematic representation of cetuximab-AF647 conjugation to silica-COOH NPs mediated by EDC coupling chemistry. (B) dSTORM imaging of silica-cetuximab-AF647 NPs of 50, 100, and 150 nm radius. TIRF microscopy images were added for comparison. Scale bar 500 and 100 nm in dSTORM zoom. (C) Scatterplot illustrating the number of cetuximab per NP versus NP radius measured by dSTORM. Color code represents NP radius according to the manufacturer. (D) Scatterplot illustrating the density of cetuximab per NP (cetuximab/ μm^2^) versus NP radius. Color code is the same as that in (C).

To investigate the conjugation efficiency of cetuximab to silica NPs, different NP sizes (50, 100, and 150 nm radius as specified by the manufacturer) were used. Subsequently, we imaged and quantified the total number of antibodies on the NP surface by dSTORM (Figure 2). Therefore, cetuximab was labeled with the dSTORM compatible dye Alexa Fluor 647 (AF647) and conjugated through lysine groups to silica NPs containing carboxylic acid surface groups using carbodiimide-based coupling chemistry, namely 1-ethyl-3-(3-(dimethylamino)propyl)-carbodiimide (EDC) (Figure 2A). Figure 2B shows representative dSTORM images obtained from cetuximab imaging on the NP surface after chemical conjugation. dSTORM imaging results in a pointillistic image, where each dot represents a detected localization originating from a stochastic detection of a single dye (Figure 2B). Due to the improved resolution of dSTORM compared to the low-resolution total internal reflection fluorescence (TIRF), the sizes of the different NP formulations can be successfully distinguished. Specifically, the properties of NPs smaller than the diffraction limit of light (250–300 nm) can be studied. The dSTORM localizations were counted around each silica NP center position using a custom clustering script, as previously described.^23^ The total number of antibodies per NP was obtained using a calibration that accounts for the stochastic appearances of one dye throughout the image acquisition (30000 frames/image) (Figure S4 in the Supporting Information).^23^ A control sample was measured with cetuximab addition but no coupling agent to determine the unspecific adsorption of cetuximab to the NP surface (Figure S5 in the Supporting Information). The results show that a fraction of antibodies adsorbed to the NP surface when no EDC was present. However, the majority of antibodies were covalently bound.

The scatter plots in Figure 2C and 2D display the total number of cetuximab per NP and the density of cetuximab per NP versus the NP radius, respectively. Since each dot in the plot represents a single NP, the interplay between NP size and the number of total antibodies can be appreciated. This feature allows the quantification of heterogeneity of the two parameters simultaneously with a single measurement.

The measured radius by dSTORM illustrates the increase associated with the antibody size (around 4–15 nm increase in NP radius depending on the antibody orientation^24^) and the size dispersion of the NPs themselves (Figure S6 and Table S3 in the Supporting Information). As expected, the number of cetuximab per NP increases with increasing NP radius at the same cetuximab/COOH group ratio. The coefficient of variation (CV) was calculated to determine the heterogeneity of cetuximab and the NP radius. The CV ranged from 20% to 60% for the number of cetuximab and from 10% to 12% for the NP radius (Figure S7 and Table S4 in the Supporting Information). These results suggest that, besides the NP size, additional sources of variability may play a role in the observed cetuximab heterogeneity. Specifically, the 150 nm radius particles present two distinct populations of particles with different ligands while the NP radius remains comparable (Figure 2C, orange circles).

To normalize the amount of cetuximab to the NP size, we calculated the cetuximab density (cetuximab/μm^2^) (Figure 2D). While 100 and 150 nm radius particles were similar in density, it almost doubled in the 50 nm radius NPs. In the latter case, the density of cetuximab slightly increases with decreasing NP radius. This difference might be attributed to the increased NP curvature at small NP sizes and COOH density variability at different NP sizes. Reports on the correlation between NP size and protein adsorption or conjugation are conflicting in the literature, but the coupling process could drive the observed differences in antibody density.^25^

We hypothesize that the variability in antibody density and the broad distributions of each formulation are due to the variability of particle size, differences in carboxyl group distribution on the NP surface, and the stochastic nature of the EDC conjugation.^5,26^ Previously, single-molecule imaging based on stepwise photobleaching pointed toward the size dispersion of liposomes as the main contributor to the distribution of ligands per NP.^13^ In line with our observations, two recent SMLM studies found that the heterogeneous particle functionalization was caused by a combination of size dispersion and nonuniform functionalization.^27,28^ Here, the single-molecule information obtained from dSTORM is a valuable tool to reveal the underlying heterogeneity of cetuximab-functionalized NPs. Additionally, different NP size ranges were imaged and quantified. In previous studies, the importance of NP size in cell uptake and toxicity is highlighted.^3,29^ Notably, the NP characterization using dSTORM proves to be a versatile tool to quantify the antibody conjugation in broad NP size ranges.

### Functionality of Silica-Cetuximab NPs is Highly Affected by a Non-Oriented Conjugation Strategy

**Figure 3:**
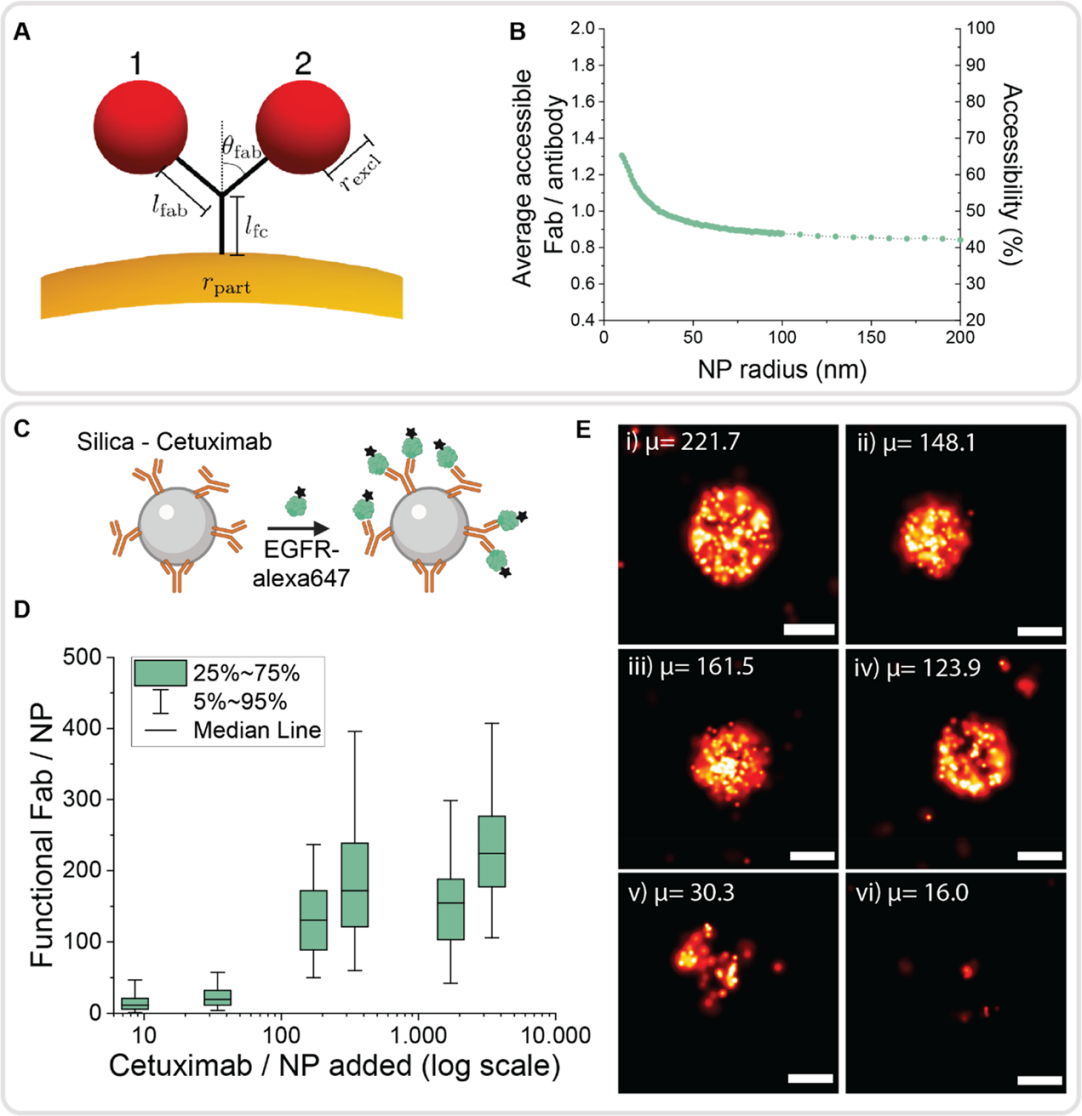
Functionality of silica-cetuximab NPs calculated using a geometrical model (A–B) and measured experimentally using a labeled EGFR probe (C–E). (A) Schematic representation of the geometrical model to describe the functionalization of cetuximab to NPs. The parameters of the model are the radius of the NP *r*_part_, the length of the stalk *l*_fc_ (5 nm), the length of the Fab domain *l*_fab_ (5 nm), and the angle that the Fab domains have with respect to the vertical θ_fab_ (0.7 rad). The NP is shown in orange, the cetuximab is shown in black, and the exclusion zones of the Fab domains are shown in red. There are two Fab domains defined 1 and 2. (B) Average accessible fab sites and accessibility (%) as a function of NP radius *r*_part_ for a random NP conjugation method (note that full accessibility corresponds to 2 Fab/NP). (C) Schematic representation of experimental NP functionality assay. Silica-cetuximab NPs were incubated with an AF647-labeled EGFR probe for detection of functional Fab fragments. (D) Functionality of silica-cetuximab NPs of 100 nm radius determined by dSTORM. Functional Fabs per NP were measured on a single-particle level for different amounts of cetuximab added during the chemical conjugation. (E) dSTORM images of EGFR-labeled silica-cetuximab NPs. Mean functional Fab/NP (μ) are indicated for each formulation. Formulations correspond to (i) 3437, (ii) 1719, (iii) 344, (iv) 172, (v) 34.37, and (vi) 8.59 Cetuximab/NP added. Scale bar 100 nm.

Carbodiimide based conjugation of antibodies to NPs is a nonoriented immobilization reaction due to the abundant primary amines on the antibody surface. Thus, some antibodies are expected to orient in an unfavorable conformation, causing reduced target-recognizing abilities. For instance, this can occur when the NP surface occludes the Fab regions.^30^ To estimate the number of accessible antibodies, we developed a geometrical model that considers a random orientation of antibodies during the NP conjugation (Figure 3A and B). A detailed explanation of the model is available in the Supporting Information. Briefly, we assumed (1) a random deposition of cetuximab, with no preferred orientation on the NP surface, and (2) a sufficiently low overall surface coverage to exclude steric hindrance between neighboring bound cetuximabs. The accessibility of cetuximab Fab regions (two per antibody) was calculated by assuming an “exclusion zone” around each Fab, representing the space that must be freely accessible for the probe to bind. Conversely, if the NP surface occludes any part of this exclusion zone no binding is possible, and we defined the Fab as inaccessible (Figure S1 in the Supporting Information). We chose a radius (*r*_excl_) of 5 nm for the exclusion zone, representing the approximate size of the EGFR probe used in the experiments (Figure 3A).

We found that the accessibility of Fab regions (*f*_A_) depends on the NP radius and that only about 40% of all conjugated Fabs is actually accessible for NPs with a radius of around 100 nm (Figure 3B). This substantial (60%) loss of potential functionality is due to geometrical orientation alone and illustrates that the number of functional conjugated antibodies may differ considerably from the total number of antibodies present on an NP surface. Previously, a mathematical model was described to study the orientation of antibodies on flat surfaces, emphasizing the poor accessibility of randomly conjugated antibodies.^31^ Consequently, characterization techniques focused on ligand functionality are desirable to understand the relationship between functional ligands and NP uptake in an experimental setting.

Taking advantage of the single-molecule resolution of dSTORM, we developed an experimental assay to count the functional cetuximabs after NP conjugation. Therefore, we required a selective labeling approach to achieve the visualization of only the functional cetuximabs. We exploited the targeting interaction to detect functional cetuximabs using a recombinant version of the EGFR as a labeling probe (Figure 3C). The probe consists of the soluble extracellular portion of the EGFR labeled with AF647 for dSTORM detection. Using this approach, only the accessible Fab will capture the EGFR probe and be imaged by dSTORM, while the occluded or nonfunctional Fab will remain undetected. The main advantage of this approach is that there is no need to label the antibody directly, preserving its native state for conjugation to NPs.

We varied the amount of cetuximab added per 100 nm radius NP (between tens to a few thousand cetuximabs/NP) to study the optimal NP ligand density needed to target a specific cell population. Other conditions such as EDC concentration, incubation time, and buffer pH remained constant. Silica NPs were functionalized with a low or high concentration of a goat antimouse antibody as a control for no EGFR binding. The total number of functional Fab fragments was estimated using a calibration as previously described for labeled cetuximab (Figure S8 in Supporting Information).

In general, we observed broad distributions of functional Fab fragments in the same NP formulation (Figure 3D and Figure S9 in Supporting Information). In fact, the average number of ligands in each formulation is not representative of the entire NP population, and an overlap between the number of ligands between different formulations is present. Furthermore, we observed that, for NPs with higher amounts of cetuximab, the distributions present longer tails corresponding to highly functional NPs. Consequently, high heterogeneity in NP functionality and targeting capabilities are expected.^32^ On the contrary, for NPs with the lowest cetuximab concentration, the EGFR binding is similar to the control NPs functionalized with antimouse antibodies (Figure S10 in Supporting Information). In contrast, the binding to bare silica NPs was considered negligible (Figure S10 in Supporting Information). When increasing the concentration of cetuximab added during NP conjugation above a certain threshold (a few hundred cetuximab/NP), we observed only a slight increase in functionality that follows a nonlinear trend. For example, adding 20 times more cetuximab per NP translated to only two times more functional Fab on average (Figure 3D and Table S5 in Supporting Information).

To demonstrate the feasibility of the developed experimental assay in other types of nanomaterials, we imaged the functionality of cetuximab-conjugated poly lactic-*co*-glycolic acid (PLGA) NPs. PLGA NPs were formulated with a 30% COOH content via nanoprecipitation, and a hydrodynamic radius of 66.4 nm was obtained (Figure S11 in Supporting Information). Subsequently, PLGA NPs were functionalized with cetuximab antibodies, and their functionality was quantified by dSTORM (Figure S12 in Supporting Information). We observed a low number of functional Fab in this nanoparticle type, thus demonstrating that fewer than tens of ligands can also be quantified with minimal unspecific binding using dSTORM.^4,5^ The results show that dSTORM is a versatile tool to quantify the functionality of antibody-conjugated NPs composed of different materials at a single-molecule level. In contrast to ensemble techniques, information regarding the single-particle functionality can be obtained, highlighting the considerable heterogeneity that can be present in antibody-conjugated NP formulations.

The functionality of cetuximab Fabs can be compromised by steric hindrance between antibodies, protein structural changes, or, as discussed earlier, unfavorable orientation on the NP surface.^24,33^ Steric hindrance between cetuximabs is not expected to play a significant role in the loss of functionality, as even at the higher cetuximab concentration saturation was not reached (estimated surface coverage between 14% and 49%). Thus, unfavorable cetuximab orientations from the EDC-mediated chemical conjugation are likely the most prominent factor. We adapted our geometrical model to assess how many cetuximabs would be accessible theoretically with an oriented conjugation approach (i.e., Fc-mediated conjugation via protein G interaction). We found that the accessibility of cetuximab Fab fragments could be improved, typically, from around 40% (nonoriented approach) to around 70% for 100 nm radius NPs (Figure S3 in Supporting Information). In line with this estimation, Saha and co-workers reported merely 20% to 30% functional antibodies conjugated via EDC-mediated reaction to 250 nm radius particles using ensemble measurements.^9^ To improve the accessibility of ligands conjugated to NPs, research has been focused on controlling NP ligand density and orientation.^11,34−36^ However, methods to determine the number of functional ligands per NP are scarce.

In combination with modeling, we demonstrate the importance of antibody orientation on the nanoparticle surface and emphasize the low fraction of functionality after nonoriented conjugation strategies. We show that dSTORM can be used to quantify the functionality of antibody-conjugated nanoparticles experimentally at a single-particle level, proving a valuable tool to assess new conjugation protocols that aid in the oriented antibody conjugation. Furthermore, the developed single-molecule characterization tool can be applied to nanomaterials of different types and sizes. Here, we demonstrate the applicability of functional dSTORM characterization in silica and polymeric PLGA NPs.

### dSTORM Imaging of EGFR Reveals Inter- and Intracellular Heterogeneities

The second parameter involved in targeting is the cell receptor density. It is known that various receptors chosen for targeted therapies (including EGFR) can also be expressed to a certain extent in healthy tissue.^37^ Therefore, a good understanding of receptor density numbers could aid in minimizing off-target effects in NP drug delivery.

To overcome these off-targeting effects, we studied the expression of EGFR in three different breast cancer cell lines known from the literature to be low (MCF-7), moderate (MDA-MB-231), and high (MDA-MB-468) expressing EGFR.^38^ To understand targeting selectivity toward these cells, we were interested in counting the density of receptors (i.e., molecules per square micron) instead of relying on qualitative trends. Therefore, we extended our functional labeling approach to map cell receptors. In this case, we used cetuximab to stain EGFR on the cell surface, which has the advantage of mapping and counting the potential binding sites of silica-cetuximab NPs (Figure 1C). Cetuximab antibodies were detected using a secondary AF647-labeled antimouse antibody recognizing the murine cetuximab Fab region. To get quantitative information on the expression profiles, we analyzed the receptor densities using dSTORM. To translate the number of dSTORM localizations to the number of receptors, we used a calibration based on low-concentration cetuximab staining on MDA-MB-468 cells to obtain isolated labeled receptors (Figure S13 in Supporting Information).^22,39^

**Figure 4:**
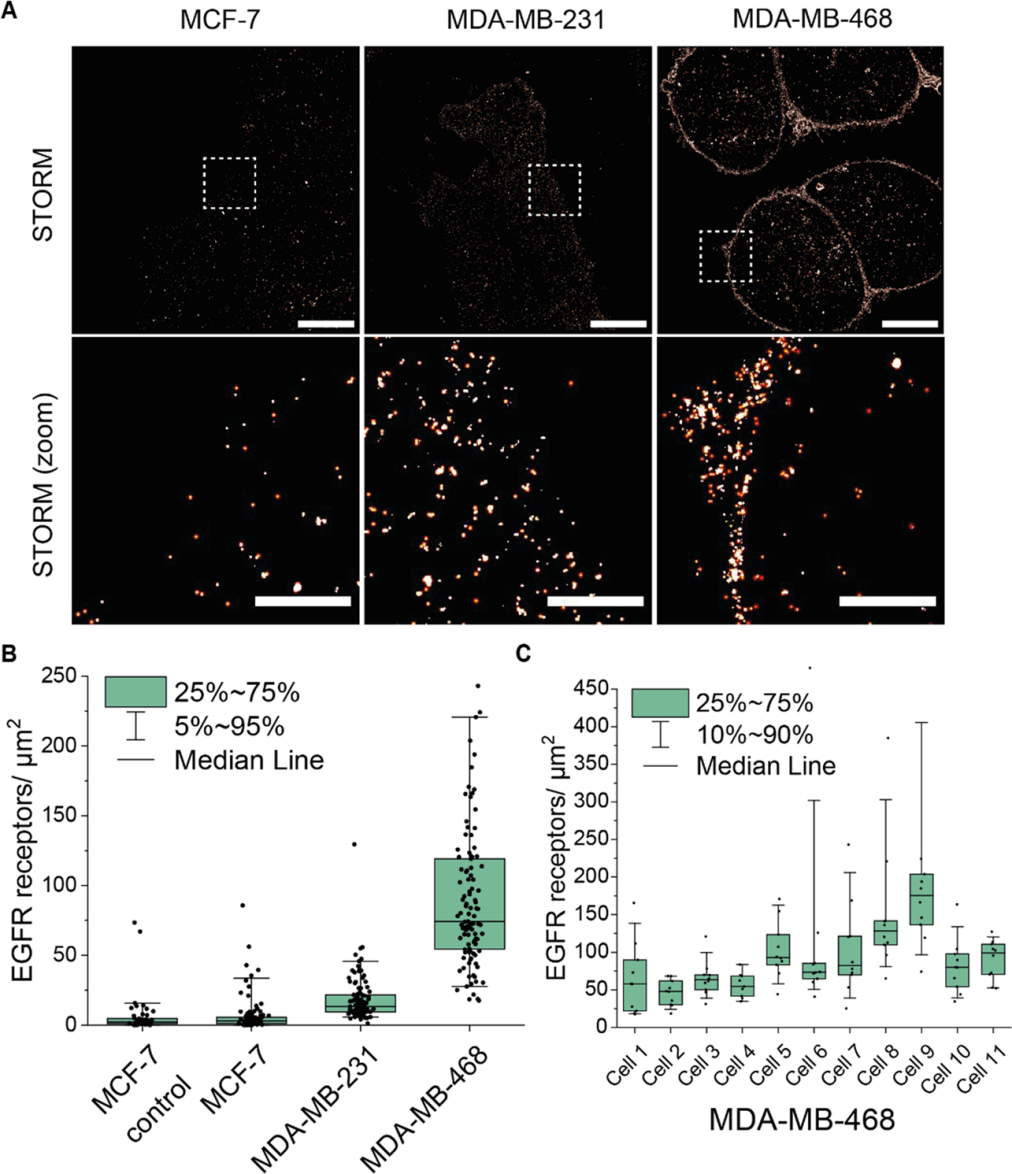
EGFR expression profiles of breast cancer cell lines MCF-7, MDA-MB-231, and MDA-MB-468. (A) Representative dSTORM images of EGFR receptors stained with cetuximab and antimouse-AF647 antibody of MCF-7, MDA-MB-231, and MDA-MB-468 cells. Scale bar indicates 10 and 2 μm for zoom in. (B) Box plots of the quantification of EGFR receptors per μm^2^. Box represents the 25% to 75% percentile and whiskers the 5% to 95%. Each data point represents one measured region of interest within a cell. In total, minimum 10 cells were measured per cell type, and for each cell 10 regions of interest were measured. MCF-7 control condition represents cells stained with secondary antibody only for unspecific binding control. (C) Variability of EGFR expression/μm^2^ between cells measured in MDA-MB-468 cells. Box represents the 25% to 75% percentile and whiskers the 10% to 90%. *Y*-axis in (B) and (C) are shortened for representation convenience, excluding single individual data points from the figure.

Representative dSTORM images of EGFR in MCF-7, MDA-MB-231, and MDA-MB-468 breast cancer cells are displayed in Figure 4A. MDA-MB-468 cells presented the highest expression level of the three cell lines, followed by MDA-MB-231 cells and MCF-7 cells, respectively (Figure 4B). This trend is in accordance with previous literature reports.^38^ Counting of receptors showed that MDA-MB-468 cells present 5.5 times higher EGFR density than MDA-231 cells. Control staining using secondary antibody only was considered negligible for both cell lines (Figure S14 in Supporting Information). The density of EGFR in MCF-7 cells was comparable to the control condition using secondary antibody labeling only (Figure 4B), indicating no detectable EGFR with our quantification method.

The single-molecule counting property of dSTORM can additionally reveal interand intracellular heterogeneities. Therefore, the density of EGFR receptors was measured per cell in 10 different regions of interest (Figure 4C). In the case of MDA-MB-468, not all cells present the same EGFR cell density. Furthermore, the EGFR distribution is heterogeneous throughout the entire cell, with changes in density depending on the location of the measured region. Spatial heterogeneity of EGFR receptors was previously described in live-cell single-molecule imaging, in accordance with the observed results.^40^ Here, dSTORM microscopy allows quantifying receptor densities and reporting single-molecule data beyond qualitative trends.^6^ Counting individual receptors bridges the limitations of current diagnostic tools, which are often semiquantitative at best. In our approach, we labeled receptors with the same antibody used in the silica-cetuximab targeting, thus imaging the potential NP binding sites.

### Selectivity and Specificity of Silica-Cetuximab Cell Uptake

**Figure 5:**
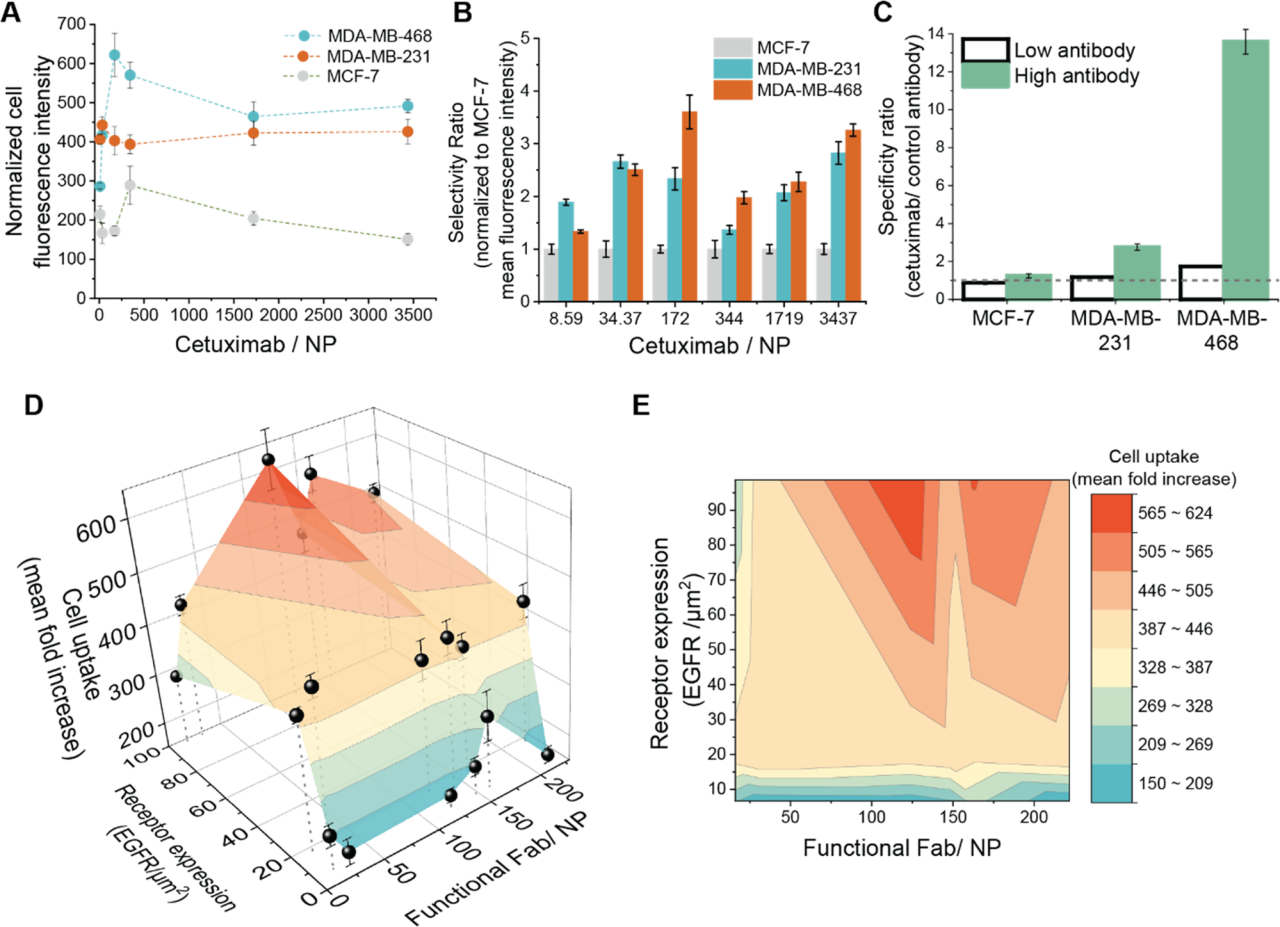
Targeting properties of silica-cetuximab NPs (100 nm radius) to breast cancer cell lines measured by flow cytometry. (A) Normalized cell fluorescence intensity after 90 min of silica-cetuximab incubation at different cetuximab conjugation amounts. Mean fluorescence intensity was normalized with respect to cells without NPs. (B) Selectivity ratio of silica-cetuximab uptake normalized to MCF-7 cells. (C) Specificity ratio of silica-cetuximab at low (34.37 antibodies/NP) and high (3427 antibodies/NP) antibody conjugation compared to control antibody conjugated NPs. A line was added for visualization purposes at specificity = 1 (no difference compared to control antibody NPs). At 0.05 significance level, the specificity ratio of MDA-MD-231 compared to MDA-MD-468 cells at low antibody conjugation is statistically significant according to a two-sample *t* test. (D) 3D visualization of the structure-targeting relationship of silica-cetuximab NPs. Color code represents mean fold fluorescence increase with respect to cells without NP (color scale presented in image E). The receptor density and NP functionality were quantified at a single-molecule level, while the cell uptake was quantified at a single-cell level. (E) 2D projection of data presented in (D).

The number of silica-cetuximab NPs internalized or firmly bound to the cell membrane was quantified by flow cytometry to study the uptake selectivity in the chosen breast cancer cell lines (Figure 5). We defined two parameters to evaluate the NP uptake, namely the selectivity and specificity ratio (Figure 5B and C, respectively). To determine the selectivity ratio, NP uptake was normalized to MCF-7 uptake, representative for low or no EGFR expression where NP uptake is expected to be minimal compared to high-expressing EGFR cells. In contrast, the specificity ratio was calculated by normalizing silica-cetuximab NP uptake to control NPs functionalized with a control antimouse antibody to account for unspecific binding of antibody-functionalized NPs.

In MDA-MB-468 and MDA-MB-231, the uptake is 2- to 4-fold higher than MCF-7 cells in most formulations (Figure 5B). The selectivity toward MDA-MB-468 cells was generally higher compared to MDA-231 cells. The highest differences between these two cell types become apparent at intermediate cetuximab concentrations. In contrast, at the higher cetuximab concentrations, the difference in selective uptake shrinks. These results suggest that there is no significant increase in uptake above a certain antibody threshold. We found that above a few hundreds of antibodies the uptake does not drastically increase further (Figure 5B).

We found that the uptake of silica-cetuximab in MCF-7 cells is close to the control formulation uptake and mainly unspecific (Figure 5C). This makes MCF-7 a good control cell line for no EGFR-specific NP uptake, as reported previously.^41^ NP uptake in MDA-MD-231 cells at low antibody concentration was mainly unspecific, while in high-expressing EGFR cells MDA-MB-468 low cetuximab functionalized NPs showed already 2-times higher specificity compared to the control formulation (Figure 5C). At high antibody concentration, functionalization of NPs with cetuximab cell uptake considerably improves in MDA-MD-231 and MDA-MB-468 cells compared to the control antibody formulation. There is a 3-fold increase in specificity of cetuximab-silica formations in MDA-MD-231 cells at high antibody concentration and a 14-fold increase in MDA-MB-468 cells, representing the highest specificity in the uptake experiment (Figure 5C).

To evaluate the influence of serum proteins on NP uptake, a similar NP uptake experiment was performed in the presence of 10% FBS (S15 in Supporting Information). In general, the normalized cell fluorescence intensity decreased between 10 and 20 times compared to NP uptake without FBS (Figures 5A and S15A in Supporting Information). At the same time, selectivity toward MDA-MB-231 and MDA-MB-468 cells increased up to 3.5 times compared with MCF-7 cells, indicating that 10% FBS reduces the unspecific binding in low EGFR-expressing cells (Figures 5B and S15B in Supporting Information). Finally, the specificity of NPs increased for low antibody concentrations but was slightly reduced at high antibody concentration when targeting MDA-MB-468 cells (Figures 5C and S15C in Supporting Information).

### Unveiling the Structure-Targeting Relationship of Silica-Cetuximab NPs

To understand the structure-targeting relationship of silica-cetuximab NPs, we correlated the number of functional Fab and the cell receptor density with the cell uptake (Figure 5D and 5E). To correlate these parameters, the NP uptake without the presence of serum proteins was considered, consistent with the conditions of the NP functional characterization. The selectivity of silica-cetuximab can be seen at (1) high Fab amounts and (2) high receptor expression, emphasizing that both parameters play a fundamental role in active targeting. Combining highly functional antibodies and high receptor expression gives a higher chance of interaction between the antibody–receptor pair. Additionally, multivalent interactions may occur in these conditions when more than one antibody interacts with more than one EGFR of the targeted cell.^42,43^

Both NP functionality and cell receptor expression thresholds are essential to design NPs that selectively target a specific receptor density. In NP functionality, we observed that there is no drastic increase in uptake above 100 functional Fab/NP in MDA-MB-231 and MDA-MB-468 cells. This observation could indicate that antibodies above this threshold are redundant for selective NP uptake. Similarly, Wang et al. reported that 25% of targeting ligand conjugated to NPs could have a similar uptake compared to 100% targeting ligand conjugation.^44^ In fact, usually more ligands than necessary are used in NP targeting,^11^ which often can have an adverse effect due to ligand crowding.^45^ It is also hypothesized that the occupation of receptors by high-density ligand NPs might decrease receptor availability and eventually lead to receptor saturation.^32^ In the case of antibody conjugation, adverse effects can also arise due to the exposure of immunogenic Fc fragments, increasing the clearance rate of those NPs.^19^ Antibody Fab fragments or nanobodies are an attractive alternative to circumvent this adversity^46^ in addition to being compatible with the presented functional dSTORM imaging.

Similarly, we observed that a specific threshold of EGFR density needs to be exceeded to obtain selective NP targeting. While MCF-7 NP uptake is mainly unspecific (mean 7 EGFR/ μm^2^), we can see specific NP uptake in MDA-MD-231 (mean 18 EGFR/ μm^2^). NP uptake is further increased at the high EGFR density MDA-MD-468 cells (mean 99 EGFR/ μm^2^), especially for high antibody functionality. In contrast, the NPs are not selective enough to differentiate between intermediate and high EGFR densities at low antibody functionality.

The understanding of the NP functionality and EGFR density thresholds is relevant to designing selective NPs. Previously, the lower and upper limits of HER2 receptor expression and NP ligand densities were explored, emphasizing that it is crucial to understand the relationship between the two parameters for targeting.^43,47^ However, the underlying heterogeneities of both parameters are often masked by reporting average values only resulting from ensemble characterization techniques.^48^ Specifically, the NP ligand coverage or functionality is mostly reported as an average of the NP population, which disregards the effect of the upper and the lower extremes of ligand functionalization in heterogeneous NP batches.

In our approach, both the NP ligands and EGFRs were quantified at a single-molecule level using dSTORM, providing a better understanding of the functional heterogeneity of antibody-conjugated NPs and EGFR expression in cells. An important feature in our experimental design is the characterization of NPs and cells with the complementary probe (EGFR or cetuximab, respectively). This characterization allowed ultimately for the structure-relationship correlation of functional NP populations and EGFR expression profiles to the observed NP uptake measured with flow cytometry at a single-cell level.

An interesting future outlook is the study of antibody functionality after the exposure to serum proteins, which will provide more insights about the NP targeting ability in complex biological environments. We show that the presence of serum proteins has a direct impact on the NP uptake. We observed that the total NP uptake is significantly lower in the presence of 10% FBS in the incubation medium. At the same time, selective NP binding toward moderate and high expressing EGFR cells could be increased. To directly correlate the NP uptake with the possible loss of NP functionality, the developed dSTORM method could be extended to characterize the functionality loss in the presence of serum proteins or after the formation of a biomolecular corona.

## Conclusions

To understand and achieve selective NP targeting, it is crucial to have quantitative information on the number of functional ligands on the NP surface and the cell receptor expression to be targeted. The nanoscale of ligands and receptors makes targeting a molecular problem. Thus, methods to quantify with single-molecule resolution are highly desired. In this work, we used dSTORM to quantify the number of accessible cetuximab antibodies conjugated to silica NPs and the density of EGFR receptors on different breast cancer cell lines at a single-molecule level. We developed a functional labeling approach, where the targeting interaction (cetuximab-EGFR) was used as a labeling tool. dSTORM imaging provided distributions of NP functionality and receptor expression, elucidating the underlying heterogeneities of both parameters. The single-molecule features are impossible to obtain using ensemble methods, which provide average values only. The antibody functionality on NPs was estimated using a geometrical model and appears to be merely 40% functional after nonoriented chemical conjugation. Experimentally, the functionality of the NPs could be assessed at different conjugated cetuximab concentrations using the extracellular domain of the EGF receptor as an imaging probe. We found that instead of the total number of antibodies, the distributions of functional antibodies are crucial to understanding the targeting activity of antibody-functionalized NPs. Furthermore, the receptors were labeled with cetuximab, thus mapping the receptor distribution that the cetuximab-conjugated NPs will encounter. Thresholds of NP functionality (a few hundred cetuximab/NP) and receptor density (a few tens EGFR/ μm^2^) were quantified by combining the single-molecule information with NP uptake studies. We found that both parameters are essential to determine the effective range of selective NP targeting. Here, the molecular mapping of functional ligands and receptors provided by dSTORM enabled an understanding of the nanoscale spatial distribution behind active targeting. We believe that the presented approach serves as an essential tool in nanomedicine characterization, aiding in the rational design of active targeted nanomedicines.

## Experimental Section

### Materials

Fluorescent silica NPs (sicastar-greenF) with surface carboxylic acid groups (COOH) of 50, 100, and 150 nm radius were purchased from Micromod Partikeltechnologie GmbH. Cetuximab antibody (Erbitux, Merck) was kindly provided by Prof. Marteen Merkx (Eindhoven University of Technology). Human EGFR protein (Fc tag, ACROBiosystems EGR-H5252), Zeba desalting columns (7K MWCO), Alexa Fluor 647 NHS ester, DMEM (high glucose, no phenol red), Penicillin-Streptomycin, Fetal Bovine Serum (qualified), Trypsin-EDTA (0.5%), HEPES buffer (1 M), and Vybrant DiO solution and Nunc cell culture flasks were obtained from Thermo Fisher Scientific. Phosphate buffered saline tablets, 1-ethyl-3-(3-(dimethylamino)propyl)-carbodiimide (EDC), tris(hydroxymethyl)-amino-methane, bovine serum albumin (96% purity), cysteamine, catalase from bovine liver, glucose oxidase, and formaldehyde 37% were purchased from Sigma-Aldrich. Sodium bicarbonate was purchased from Merck. Sodium chloride was purchased from Sanal. MDA-MB-231 and MCF-7 cells were kindly provided by Prof. Jaap den Toonder (Eindhoven University of Technology). MDA-MB-468 cells were obtained from ATCC (HTB-132). Alexa Fluor 647 AffiniPure Goat Anti-Mouse antibody and plain AffiniPure Goat Antimouse antibody were purchased from Jackson Immunoresearch. μ-slide 8-well glass bottom chambered coverslips (#1.5H) were obtained from Ibidi. Poly(lactide-*co*-glycolide) AP082 (Mn 25000–35000) and Poly(lactide-*co*-glycolide)-*b*-poly(ethylene glycol)-carboxylic acid end-cap AI078 (PLGA-PEG-COOH, Mw 20:5 kDa) were purchased from Akina Inc. Poly(lactide-*co*-glycolide)-methoxy-poly(ethylene glycol) (Mw PLGA:PEG, 30:1 kDa, L:G in PLGA 50:50) was supplied from Biochempeg Scientific Inc.

### Labeling of Cetuximab and EGFR Protein

Prior to fluorescent labeling, cetuximab was buffer exchanged to sodium bicarbonate (pH 8.4 0.1M) using a Zeba desalting colum. Cetuximab and EGFR protein were incubated with Alexa Fluor 647 NHS ester at a 1:8 mol and 1:5 mol ratio protein/dye, respectively, for 2 h at 22 °C and 400 rpm in a ThermoMixer (Eppendorf). The reaction mixture was purified using two consecutive Zeba desalting columns rinsed with PBS buffer according to the manufacturer’s protocol. The UV–vis of the final products were measured to calculate the degree of labeling with using a NanoDrop One (Thermo) with PBS as the blank measurement. For cetuximab-AF647 and EGFR-AF647, degrees of labeling of 5.4 and 2.4 were obtained, respectively.

### Conjugation of Cetuximab to Silica-COOH NPs

Cetuximab or cetuximab-AF647 was conjugated to silica-COOH NPs in MES buffer (50 mM, pH 5) via 1-ethyl-3-(3-(dimethylamino)propyl)-carbodiimide (EDC) coupling chemistry. First, NPs were washed in 500 μL of MES buffer and centrifuged 10 min at 16 000*g*. NPs were resuspended in MES buffer (50 mM, pH 5) containing 2 mM EDC and incubated for 15 min at 22 °C and 400 rpm in a ThermoMixer. NPs were then sonicated for 5 min in a bath sonicator. Next, cetuximab antibody was added to the EDC activated NPs at the desired concentration and incubated for 2 h at 22 °C and 400 rpm in a ThermoMixer. To determine the unspecific cetuximab binding, the same reaction was performed without EDC activation. To conjugate cetuximab antibody to NPs of different sizes, the cetuximab/COOH and EDC/COOH ratio was kept constant (0.68 cetuximab/COOH and 1963 mol EDC/mol COOH). For the concentration range of antibodies, the ratio was kept between 8.6 and 3437 antibodies/NP and the 100 nm radius NPs were used (Table S5 in the Supporting Information). As a control formulation, silica-COOH NPs of 100 nm radius were incubated with a goat antimouse antibody at low (8.6 antibodies/NP) and high (3437 antibodies/NP) concentrations. Unconjugated antibody was purified by washing with 25 mM HEPES buffer and centrifuging thrice at 16 000*g* for 15 min. Silica-cetuximab NPs were resuspended at a final concentration of 1 mg/mL in 25 mM HEPES buffer and stored at 4 °C.

### Incubation of Silica-Cetuximab NPs with EGFR-AF647 Probe

The functionality of cetuximab antibodies conjugated to silica NPs was studied by quantifying the number of EGFR-AF647 probes bound to each NP. Silica-cetuximab NPs were first sonicated in a bath sonicator for 10 min. Next, 25 μL of NPs (1 mg/mL) were incubated with 20 pmol of EGFR probe and 0.5% bovine serum albumin to block unspecific binding for 1 h at 25 °C and 400 rpm in a ThermoMixer. NPs were sonicated in a bath sonicator for 5 min to aid redispersion and imaged the same day.

### Optical Setup

dSTORM imaging was performed with a Nikon N-STORM system configured for TIRF imaging and equipped with a perfect focus system. AF647-labeled proteins were illuminated using a 647 nm laser (170 mW), and sicastar-greenF NPs were illuminated using a 488 nm laser (90 mW) with an adjusted TIRF angle to maximize the signal-to-noise ratio. No UV activation was used. A Nikon 100X, 1.4 NA oil immersion objective was used to collect the fluorescence signal, which was passed through a quad-band-pass dichroic filter (97335, Nikon) and recorded on an Andor EMCCD camera (ixon3) with pixel size 160 nm and a region of interest of 256 × 256 pixels.

### dSTORM Imaging of NPs

Coverslips (22 mm × 22 mm, #1.5) were sonicated in isopropanol for 20 min and dried under nitrogen flow, and microscope slides (76 mm × 26 mm, thickness 1 mm) were cleaned using an isopropanol-soaked tissue before each experiment. An imaging chamber was prepared by attaching one coverslip to a microscope slide using double-sided scotch tape. This created a chamber of approximately 20 μL volume. Silica-cetuximab or silica-cetuximab-EGFR NPs were incubated in the imaging chamber and allowed to adsorb for 20–30 min at room temperature. The imaging chamber was rinsed with 200 μL of HEPES buffer (25 mM) to remove nonattached NPs and subsequently rinsed with 100 μL of STORM buffer^49^ (50 mM Tris pH 8, 10 mM NaCl, 10% w/v glucose, 50 mM cysteamine, 0.5 mg/mL glucose oxidase, 40 μg/mL catalase). Flow chambers were sealed with nail polish to prevent solvent evaporation. TIRF images of the 488 and 647 channel were acquired before dSTORM at 2% laser power and 100 ms exposure. For dSTORM, samples with cetuximab-AF647 and EGFR-AF647 were acquired for 30 000 frames at 30 ms exposure time and 100% laser power for the 647 channel. The fluorescent silica NPs were used to identify the NP position and drift correction of the final image by collecting one frame every 100 frames in the 488 channel at the same integration time and 5–10% laser power. A minimum of 100 NPs were imaged for each condition in 2 to 4 different fields of view. To estimate the number of blinks per cetuximab-647 or EGFR-647 protein, a calibration was performed under the same imaging conditions at very low protein concentration (8.76 pM and 4.32 pM, respectively) attached to a cover glass.

### dSTORM Analysis of NPs

dSTORM images were analyzed with the Nikon NIS elements software (version 5.21.01). dSTORM localizations were detected by Gaussian fitting of the blinking dyes, with a minimum intensity height threshold of 400 for the 647 channel of cetuximab-AF647 NPs, 300 for the 647 channel of EGFR-AF647 NPs, and 150 for the 488 channel in both cases. Analysis was started at frame number 400 for cetuximab-AF647 imaging and 200 for EGFR-AF647 imaging to eliminate nonblinking behavior in the first instances of the sample illumination. Molecules detected in 5 consecutive frames were counted as a single fluorophore to prevent overcounting of blinks from the same dye. Molecules detected for more than 5 consecutive frames were discarded. A density filter of minimum 10 localization in a radius of 200 nm was applied to remove the background signal originating from free dye or labeled proteins attached to the cover glass. The dSTORM localization list was imported and run through a custom MATLAB script to quantify the number of localizations for each NP. The code is extensively reported elsewhere.^23^ Briefly, a mean shift clustering algorithm was applied to cluster the 488 localizations from the silica NPs. The bandwidth was adjusted to 100 nm, and clusters with less than 20 localizations were discarded. Next, the number of 647 localizations were counted around each NP center. For 50, 100, and 150 nm radius NPs the maximum counting distance was a 130, 180, and 200 nm radius, respectively. Aggregates with an unrealistic size were filtered out. The analysis output provided the number of localizations in the 647 channel and the NP radius. The data were plotted in scatter plots or histograms using Origin 2020. Data histograms were fitted with the same software. For single protein calibration, localizations were clustered using the mean shift clustering algorithm^23^ with a bandwidth of 100 nm and a minimum of 2 points per cluster separated in a maximum radius of 15 nm. The number of blinks per single protein were plotted in a histogram, and an exponential decay function was fitted to calculate the mean number of blinks per protein using the Origin 2020 software.

### Immunostaining of EGFR in Breast Cancer Cell Lines

MCF-7, MDA-MB-231, and MDA-MB-468 cells were cultured in DMEM (high glucose, no phenol red) supplemented with 10% Fetal bovine serum and penicillin-streptomycin (100 U/mL) at 37 °C and 5% CO_2_. For imaging, cells were detached from culture flasks using trypsin and seeded at a density of 50 000 cells/well in Ibidi μ-slide 8-well glass bottom chambered coverslips. After 48 h, cells were washed once with warm PBS and fixated using 3.7% formaldehyde solution for 10 min at room temperature. After fixation, cells were washed thrice with PBS and blocked with 5% BSA solution in PBS overnight at 4 °C or 1 h at 22 °C. Primary antibody staining with cetuximab was performed for 2 h at room temperature using 10 μg/mL cetuximab and 5% BSA in a volume of 150 μL/well. Subsequently, cells were rinsed thrice with PBS and stained with a AF647 secondary antimouse antibody diluted 1:150 in PBS containing 5% BSA in a total volume of 150 μL/well and incubated for 1 h at room temperature. Cells were rinsed with PBS thrice and postfixated using 1% formaldehyde solution for 10 min at room temperature. Finally, cells were washed thrice with PBS and stored at 4 °C before imaging. As a control for unspecific binding, cells were incubated with AF647 secondary antimouse antibody only. To obtain isolated labeled EGFR receptors for STORM calibration MDA-MB-468 cells were stained at low cetuximab concentration (0.01 μg/mL), while secondary antibody concentration was maintained to be constant.

### dSTORM Imaging of Cells

Before dSTORM imaging of cells, the PBS storage solution was substituted for STORM buffer (5% w/v glucose, 100 mM cysteamine, 0.5 mg/mL glucose oxidase, 40 μg/mL catalase in PBS). Cells were acquired for 20 000 frames at 16 ms exposure time and 100% laser power for the 647 channel. Between 10 and 11 cells were imaged for each cell type and between 3 and 6 cells for each control (secondary antibody only).

### dSTORM Analysis of Cells

dSTORM images were analyzed with the Nikon NIS elements software (version 5.21.01). dSTORM localizations were detected by Gaussian fitting of the blinking dyes, with a minimum intensity height threshold of 150 for the 647 channel. Analysis was started at frame number 100 to eliminate nonblinking behavior in the first instances of the sample illumination. Molecules detected in 5 consecutive frames were counted as a single fluorophore to prevent overcounting of blinks from the same dye. Molecules detected for more than 5 consecutive frames were discarded. Drift correction was performed in the NIS elements software, based on an autocorrelation function. The dSTORM localization list was imported and run through a custom MATLAB script to quantify localizations’ density in each cell type. Ten ROIs were selected manually and stochastically per cell by drawing a polygonal area on the low-resolution fluorescent or bright field image. Finally, the density of dSTORM localizations in the defined areas were obtained. ROIs with an unrealistic number of localizations were excluded from the analysis. To determine the number of blinks per EGFR receptor, a low concentration of cetuximab staining (0.01 μg/mL) was performed on MDA-MB-468 cells to obtain isolated receptors.^39^ The resulting dSTORM localizations from these samples were analyzed with a custom MATLAB script. Localizations were clustered using the mean shift clustering algorithm described for single-protein calibration using a bandwidth of 100 nm and a minimum of 2 points per cluster. Clusters bigger than a 100 nm radius were discarded from the analysis. The resulting localizations per cluster, corresponding to isolated receptors, were plotted in a histogram, and the distribution of localizations per receptor was fitted using an exponential decay function in the Origin 2020 software to extract the mean number of localizations per receptor.

### NP Uptake by Flow Cytometry

MCF-7, MDA-MB-231, and MDA-MB-468 cells were in a 24-well plate at a density of 95 000 cells/well and incubated for 48 h at 37 °C and 5% CO_2_. Cells were washed once with PBS and incubated with different NP formulations at a final concentration of 150 μg/mL NPs in DMEM without FBS (final volume 500 μL/well) for 90 min at 37 °C and 5% CO_2_. For comparison, NP uptake was additionally performed in the presence of 10% FBS (Figure S15 in Supporting Information). Cells were washed once with PBS before detachment and centrifuged at 300*g* for 5 min. Cells were resuspended in 300 μL of BSA 1% in PBS and kept on ice before the flow cytometry measurement. For each condition, a minimum of 20 000 cells were measured on a BD FACSCanto II configured for FITC detection. Flow cytometry data were analyzed using FlowJo (version 10.7.1). The gating strategy used is shown in Figure S16 in the Supporting Information.

## Supporting Information Available

Supplementary Methods and Supplementary Figures available online. Geometrical model of antibody accessibility; synthesis, characterization and dSTORM functionality imaging of PLGA NPs; dSTORM calibration of fluorescent probes; silica-cetuximab coupling control; size dispersion of silica; cetuximab/silica NP and NP size measured by dSTORM; histograms of silica-cetuximab functionality; number of dSTORM localizations/EGFR measured in MDA-MB-468; unspecific binding control to cells; targeting of silica-cetuximab in the presence of 10% FBS; example of FACS gating strategy. (PDF)

## References

[ref1] Marques A. C.; Costa P. J.; Velho S.; Amaral M. H. Functionalizing Nanoparticles with Cancer-Targeting Antibodies: A Comparison of Strategies. J. Controlled Release 2020, 320, 180–200. 10.1016/j.jconrel.2020.01.035. 31978444

[ref2] Mitchell M. J.; Billingsley M. M.; Haley R. M.; Wechsler M. E.; Peppas N. A.; Langer R. Engineering Precision Nanoparticles for Drug Delivery. Nat. Rev. Drug Discovery 2021, 20, 101 10.1038/s41573-020-0090-8. 33277608 PMC7717100

[ref3] Dai Q.; Wilhelm S.; Ding D.; Syed A. M.; Sindhwani S.; Zhang Y.; Chen Y. Y.; MacMillan P.; Chan W. C. W. Quantifying the Ligand-Coated Nanoparticle Delivery to Cancer Cells in Solid Tumors. ACS Nano 2018, 12 (8), 8423–8435. 10.1021/acsnano.8b03900. 30016073

[ref4] Woythe L.; Tito N. B.; Albertazzi L. A Quantitative View on Multivalent Nanomedicine Targeting. Adv. Drug Delivery Rev. 2021, 169, 1–21. 10.1016/j.addr.2020.11.010. 33264593

[ref5] Rabanel J.-M.; Adibnia V.; Tehrani S. F.; Sanche S.; Hildgen P.; Banquy X.; Ramassamy C. Nanoparticle Heterogeneity: An Emerging Structural Parameter Influencing Particle Fate in Biological Media?. Nanoscale 2019, 11 (2), 383–406. 10.1039/C8NR04916E. 30560970

[ref6] Nerreter T.; Letschert S.; Götz R.; Doose S.; Danhof S.; Einsele H.; Sauer M.; Hudecek M. Super-Resolution Microscopy Reveals Ultra-Low CD19 Expression on Myeloma Cells That Triggers Elimination by CD19 CAR-T. Nat. Commun. 2019, 10 (1), 3137 10.1038/s41467-019-10948-w. 31316055 PMC6637169

[ref7] Lo Giudice M. C.; Herda L. M.; Polo E.; Dawson K. A. *In Situ* Characterization of Nanoparticle Biomolecular Interactions in Complex Biological Media by Flow Cytometry. Nat. Commun. 2016, 7 (1), 13475 10.1038/ncomms13475. 27845346 PMC5116075

[ref8] Cahall C. F.; lilly J. l.; Hirschowitz E. A.; Berron B. J. A Quantitative Perspective on Surface Marker Selection for the Isolation of Functional Tumor Cells. Breast Cancer Basic Clin. Res. 2015, 9s1, BCBCR.S25461 10.4137/BCBCR.S25461. PMC4517843 26309407

[ref9] Saha B.; Songe P.; Evers T. H.; Prins M. W. J. The Influence of Covalent Immobilization Conditions on Antibody Accessibility on Nanoparticles. Analyst 2017, 142 (22), 4247–4256. 10.1039/C7AN01424D. 29068008

[ref10] Jeong J.; Kim W.; Kim L. K.; VanHouten J.; Wysolmerski J. J. HER2 Signaling Regulates HER2 Localization and Membrane Retention. PLoS One 2017, 12 (4), e0174849 10.1371/journal.pone.0174849. 28369073 PMC5378417

[ref11] Alkilany A. M.; Zhu L.; Weller H.; Mews A.; Parak W. J.; Barz M.; Feliu N. Ligand Density on Nanoparticles: A Parameter with Critical Impact on Nanomedicine. Adv. Drug Delivery Rev. 2019, 143, 22–36. 10.1016/j.addr.2019.05.010. 31158406

[ref12] Coty J.-B.; Vauthier C. Characterization of Nanomedicines: A Reflection on a Field under Construction Needed for Clinical Translation Success. J. Controlled Release 2018, 275, 254–268. 10.1016/j.jconrel.2018.02.013. 29454063

[ref13] Belfiore L.; Spenkelink L. M.; Ranson M.; van Oijen A. M.; Vine K. L. Quantification of Ligand Density and Stoichiometry on the Surface of Liposomes Using Single-Molecule Fluorescence Imaging. J. Controlled Release 2018, 278, 80–86. 10.1016/j.jconrel.2018.03.022. 29577949

[ref14] Pujals S.; Feiner-Gracia N.; Delcanale P.; Voets I.; Albertazzi L. Super-Resolution Microscopy as a Powerful Tool to Study Complex Synthetic Materials. Nat. Rev. Chem. 2019, 3 (2), 68–84. 10.1038/s41570-018-0070-2.

[ref15] Pujals S.; Albertazzi L. Super-Resolution Microscopy for Nanomedicine Research. ACS Nano 2019, 13 (9), 9707–9712. 10.1021/acsnano.9b05289. 31424198 PMC6764015

[ref16] García-Fernández L.; Garcia-Pardo J.; Tort O.; Prior I.; Brust M.; Casals E.; Lorenzo J.; Puntes V. F. Conserved Effects and Altered Trafficking of Cetuximab Antibodies Conjugated to Gold Nanoparticles with Precise Control of Their Number and Orientation. Nanoscale 2017, 9 (18), 6111–6121. 10.1039/C7NR00947J. 28447703

[ref17] Herda L. M.; Hristov D. R.; Lo Giudice M. C.; Polo E.; Dawson K. A. Mapping of Molecular Structure of the Nanoscale Surface in Bionanoparticles. J. Am. Chem. Soc. 2017, 139 (1), 111–114. 10.1021/jacs.6b12297. 28005336

[ref18] Delcanale P.; Miret-Ontiveros B.; Arista-Romero M.; Pujals S.; Albertazzi L. Nanoscale Mapping Functional Sites on Nanoparticles by Points Accumulation for Imaging in Nanoscale Topography (PAINT). ACS Nano 2018, 12 (8), 7629–7637. 10.1021/acsnano.7b09063. 30048592

[ref19] Kappel C.; Seidl C.; Medina-Montano C.; Schinnerer M.; Alberg I.; Leps C.; Sohl J.; Hartmann A.-K.; Fichter M.; Kuske M.; Schunke J.; Kuhn G.; Tubbe I.; Paßlick D.; Hobernik D.; Bent R.; Haas K.; Montermann E.; Walzer K.; Diken M. Density of Conjugated Antibody Determines the Extent of Fc Receptor Dependent Capture of Nanoparticles by Liver Sinusoidal Endothelial Cells. ACS Nano 2021, 15, 15191 10.1021/acsnano.1c05713. 34431291

[ref20] Bates M.; Huang B.; Dempsey G. T.; Zhuang X. Multicolor Super-Resolution Imaging with Photo-Switchable Fluorescent Probes. Science 2007, 317 (5845), 1749–1753. 10.1126/science.1146598. 17702910 PMC2633025

[ref21] Nicovich P. R.; Owen D. M.; Gaus K. Turning Single-Molecule Localization Microscopy into a Quantitative Bioanalytical Tool. Nat. Protoc. 2017, 12 (3), 453–460. 10.1038/nprot.2016.166. 28151466

[ref22] Tobin S. J.; Wakefield D. L.; Jones V.; Liu X.; Schmolze D.; Jovanović-Talisman T. Single Molecule Localization Microscopy Coupled with Touch Preparation for the Quantification of Trastuzumab-Bound HER2. Sci. Rep. 2018, 8 ( (1), ). 10.1038/s41598-018-33225-0. PMC6181918 30310083

[ref23] Feiner-Gracia N.; Beck M.; Pujals S.; Tosi S.; Mandal T.; Buske C.; Linden M.; Albertazzi L. Super-Resolution Microscopy Unveils Dynamic Heterogeneities in Nanoparticle Protein Corona. Small 2017, 13 (41), 1701631 10.1002/smll.201701631. 28922574

[ref24] Welch N. G.; Scoble J. A.; Muir B. W.; Pigram P. J. Orientation and Characterization of Immobilized Antibodies for Improved Immunoassays (Review). Biointerphases 2017, 12 (2), 02D301 10.1116/1.4978435. 28301944

[ref25] Nasir I.; Lundqvist M.; Cabaleiro-Lago C. Size and Surface Chemistry of Nanoparticles Lead to a Variant Behavior in the Unfolding Dynamics of Human Carbonic Anhydrase. Nanoscale 2015, 7 (41), 17504–17515. 10.1039/C5NR05360A. 26445221

[ref26] Post R. A. J.; van der Zwaag D.; Bet G.; Wijnands S. P. W.; Albertazzi L.; Meijer E. W.; van der Hofstad R. W. A Stochastic View on Surface Inhomogeneity of Nanoparticles. Nat. Commun. 2019, 10 (1), 1663 10.1038/s41467-019-09595-y. 30971686 PMC6458121

[ref27] Lubken R. M.; de Jong A. M.; Prins M. W. J. How Reactivity Variability of Biofunctionalized Particles Is Determined by Superpositional Heterogeneities. ACS Nano 2021, 15 (1), 1331–1341. 10.1021/acsnano.0c08578. 33395272 PMC7844819

[ref28] Andrian T.; Delcanale P.; Pujals S.; Albertazzi L. Correlating Super-Resolution Microscopy and Transmission Electron Microscopy Reveals Multiparametric Heterogeneity in Nanoparticles. Nano Lett. 2021, 21, 5360 10.1021/acs.nanolett.1c01666. 34125548 PMC8227466

[ref29] Bannunah A. M.; Vllasaliu D.; Lord J.; Stolnik S. Mechanisms of Nanoparticle Internalization and Transport Across an Intestinal Epithelial Cell Model: Effect of Size and Surface Charge. Mol. Pharmaceutics 2014, 11 (12), 4363–4373. 10.1021/mp500439c. 25327847

[ref30] Puertas S.; Batalla P.; Moros M.; Polo E.; del Pino P.; Guisán J. M.; Grazú V.; de la Fuente J. M. Taking Advantage of Unspecific Interactions to Produce Highly Active Magnetic Nanoparticle–Antibody Conjugates. ACS Nano 2011, 5 (6), 4521–4528. 10.1021/nn200019s. 21526783

[ref31] Mackey D.; Kelly E.; Nooney R.; O’Kennedy R. Direct Immunoassays and Their Performance – Theoretical Modelling of the Effects of Antibody Orientation and Associated Kinetics. Integr. Biol. 2018, 10 (10), 598–604. 10.1039/C8IB00077H. 30187065

[ref32] Elias D. R.; Poloukhtine A.; Popik V.; Tsourkas A. Effect of Ligand Density, Receptor Density, and Nanoparticle Size on Cell Targeting. Nanomedicine Nanotechnol. Biol. Med. 2013, 9 (2), 194–201. 10.1016/j.nano.2012.05.015. PMC3502720 22687896

[ref33] Lo Giudice M. C.; Meder F.; Polo E.; Thomas S. S.; Alnahdi K.; Lara S.; Dawson K. A. Constructing Bifunctional Nanoparticles for Dual Targeting: Improved Grafting and Surface Recognition Assessment of Multiple Ligand Nanoparticles. Nanoscale 2016, 8 (38), 16969–16975. 10.1039/C6NR05478A. 27714073

[ref34] Colombo M.; Fiandra L.; Alessio G.; Mazzucchelli S.; Nebuloni M.; De Palma C.; Kantner K.; Pelaz B.; Rotem R.; Corsi F.; Parak W. J.; Prosperi D. Tumour Homing and Therapeutic Effect of Colloidal Nanoparticles Depend on the Number of Attached Antibodies. Nat. Commun. 2016, 7, 13818 10.1038/ncomms13818. 27991503 PMC5187442

[ref35] Mazzucchelli S.; Sommaruga S.; O’Donnell M.; Galeffi P.; Tortora P.; Prosperi D.; Colombo M. Dependence of Nanoparticle-Cell Recognition Efficiency on the Surface Orientation of ScFv Targeting Ligands. Biomater. Sci. 2013, 1 (7), 728 10.1039/c3bm60068h. 32481827

[ref36] Sivaram A. J.; Wardiana A.; Howard C. B.; Mahler S. M.; Thurecht K. J. Recent Advances in the Generation of Antibody-Nanomaterial Conjugates. Adv. Healthc. Mater. 2018, 7 (1), 1700607 10.1002/adhm.201700607. 28961378

[ref37] Cheng Z.; Al Zaki A.; Hui J. Z.; Muzykantov V. R.; Tsourkas A. Multifunctional Nanoparticles: Cost *Versus* Benefit of Adding Targeting and Imaging Capabilities. Science 2012, 338 (6109), 903–910. 10.1126/science.1226338. 23161990 PMC3660151

[ref38] Patel D.; Guo X.; Ng S.; Melchior M.; Balderes P.; Burtrum D.; Persaud K.; Luna X.; Ludwig D. L.; Kang X. IgG Isotype, Glycosylation, and EGFR Expression Determine the Induction of Antibody-Dependent Cellular Cytotoxicity *in Vitro* by Cetuximab. Hum. Antibodies 2010, 19 (4), 89–99. 10.3233/HAB-2010-0232. 21178280

[ref39] Letschert S.; Göhler A.; Franke C.; Bertleff-Zieschang N.; Memmel E.; Doose S.; Seibel J.; Sauer M. Super-Resolution Imaging of Plasma Membrane Glycans. Angew. Chem., Int. Ed. 2014, 53 (41), 10921–10924. 10.1002/anie.201406045. 25164466

[ref40] Delcanale P.; Porciani D.; Pujals S.; Jurkevich A.; Chetrusca A.; Tawiah K. D.; Burke D. H.; Albertazzi L. Aptamers with Tunable Affinity Enable Single-Molecule Tracking and Localization of Membrane Receptors on Living Cancer Cells. Angew. Chem., Int. Ed. 2020, 59 (42), 18546–18555. 10.1002/anie.202004764. PMC7590183 32627326

[ref41] Haeri A.; Zalba S.; ten Hagen T. L. M.; Dadashzadeh S.; Koning G. A. EGFR Targeted Thermosensitive Liposomes: A Novel Multifunctional Platform for Simultaneous Tumor Targeted and Stimulus Responsive Drug Delivery. Colloids Surf. B Biointerfaces 2016, 146, 657–669. 10.1016/j.colsurfb.2016.06.012. 27434152

[ref42] Hammink R.; Mandal S.; Eggermont L. J.; Nooteboom M. Controlling T-Cell Activation with Synthetic Dendritic Cells Using the Multivalency Effect. ACS Omega 2017, 2, 937 10.1021/acsomega.6b00436. 28393131 PMC5377267

[ref43] Wang J.; Min J.; Eghtesadi S. A.; Kane R. S.; Chilkoti A. Quantitative Study of the Interaction of Multivalent Ligand-Modified Nanoparticles with Breast Cancer Cells with Tunable Receptor Density. ACS Nano 2020, 14 (1), 372–383. 10.1021/acsnano.9b05689. 31899613

[ref44] Wang J.; Tian S.; Petros R. A.; Napier M. E.; DeSimone J. M. The Complex Role of Multivalency in Nanoparticles Targeting the Transferrin Receptor for Cancer Therapies. J. Am. Chem. Soc. 2010, 132 (32), 11306–11313. 10.1021/ja1043177. 20698697 PMC2923393

[ref45] Byzova N. A.; Safenkova I. V.; Slutskaya E. S.; Zherdev A. V.; Dzantiev B. B. Less Is More: A Comparison of Antibody–Gold Nanoparticle Conjugates of Different Ratios. Bioconjugate Chem. 2017, 28 (11), 2737–2746. 10.1021/acs.bioconjchem.7b00489. 28984436

[ref46] Richards D. A.; Maruani A.; Chudasama V. Antibody Fragments as Nanoparticle Targeting Ligands: A Step in the Right Direction. Chem. Sci. 2017, 8 (1), 63–77. 10.1039/C6SC02403C. 28451149 PMC5304706

[ref47] Li M.; Dong J.; Cheng F.; Li C.; Wang H.; Sun T.; He W.; Wang Q. Controlling Conjugated Antibodies at the Molecular Level for Active Targeting Nanoparticles toward HER2-Positive Cancer Cells. Mol. Pharm. 2021, 18, 1196 10.1021/acs.molpharmaceut.0c01090. 33448219

[ref48] Mullen D. G.; Banaszak Holl M. M. Heterogeneous Ligand–Nanoparticle Distributions: A Major Obstacle to Scientific Understanding and Commercial Translation. Acc. Chem. Res. 2011, 44 (11), 1135–1145. 10.1021/ar1001389. 21812474 PMC3217083

[ref49] Jimenez A.; Friedl K.; Leterrier C. About Samples, Giving Examples: Optimized Single Molecule Localization T Microscopy. Methods 2020, 174 (March 2020), 100–114. 10.1016/j.ymeth.2019.05.008. 31078795

