# Supplementary material for: A Single-Molecule View at Nanoparticle Targeting Selectivity: Correlating Ligand Functionality and Cell Receptor Density
Source: ACS Nano. 2022 Mar 11;16(3):3785–96. doi: 10.1021/acsnano.1c08277 (PMC8945370; doi:10.1021/acsnano.1c08277)
Supplement: Supplementary file 1 — nn1c08277_si_001.pdf [file nn1c08277_si_001.pdf]

A Single-Molecule View at Nanoparticle Targeting Selectivity: Correlating Ligand Functionality and Cell Receptor Density

Laura Woythe[†], Pranav Madhikar[ẟ], Natalia Feiner-Gracia[†], Cornelis Storm[ẟ], Lorenzo Albertazzi[‡,*]

†Department of Biomedical Engineering, Institute for Complex Molecular Systems (ICMS), Eindhoven University of Technology, Eindhoven 5612AZ, the Netherlands and ‡Institute for Bioengineering of Catalonia (IBEC), The Barcelona Institute of Science and Technology (BIST), Barcelona 08036, Spain

ẟDepartment of Applied Physics, Institute for Complex Molecular Systems (ICMS), Eindhoven University of Technology, Den Dolech 2, 5600MB Eindhoven, the Netherlands

*correspondence: l.albertazzi@tue.nl

Supporting information

CONTENTS

Supplementary Methods

1. Scanning electron microscopy (SEM) imaging of sicastar®-greenF NPs.
2. Geometrical model of antibody accessibility
3. Synthesis of PLGA NPs
4. Conjugation of antibodies to PLGA NPs
5. Incubation of PLGA NPs with EGFR-AF647 probe.
6. TIRF and dSTORM imaging of PLGA NPs
7. dSTORM analysis of PLGA NPs

Supplementary Figures

Figure S1. Molecular representation of EGFR bound to one of the Fab sites in Cetuximab.

Figure S2. Three angles parametrize the orientation of the Cetuximab model.

Table S1. Numerical values of the various parameters used in the model.

Table S2: Measured and theoretical maximum number of antibodies per NP as a function of NP radius.

Figure S3. Estimates of the number of accessible Fab sites.

Figure S4. Quantification of dSTORM localizations per single cetuximab antibody.

Figure S5. dSTORM quantification of cetuximab-AF647 binding to 100 nm radius silica NPs without and with the covalent coupling agent 1-ethyl-3-(3-dimethylaminopropyl)-carbodiimide (EDC).

Figure S6. Size dispersion of silica-COOH NPs measured by SEM

Table S3. Coefficient of variation (CV, %) of the NP radius measured by SEM for NPs with different radii.

Figure S7. Number of cetuximab per NP and NP size measured by dSTORM.

Table S4. Coefficient of variation (CV, %) of the number of cetuximab and NP radius measured by dSTORM for NPs with different radii.

Figure S8. Quantification of dSTORM localizations per single recombinant EGFR probe.

Figure S9. Distributions of functional Fab fragments at different added cetuximab concentrations in the conjugation reaction.

Table S5. Mean and SD of functional Fab fragments extracted from histogram distribution fittings.

Figure S10. Binding of EGFR probe to control NPs and silica-COOH NP without antibody (Plain NP).

Figure S11. Hydrodynamic diameter of formulated PLGA-COOH NPs measured by dynamic light scattering.

Figure S12. dSTORM imaging of cetuximab-conjugated PLGA NPs.

Figure S13. Number of localizations / EGFR measured in low concentration cetuximab staining of MDA-MB-468 cells.

Figure S14. Unspecific binding control of Alexa647-labeled secondary antibody to MDA-MB-231 and MDA-MB-468 cells.

Figure S15. Targeting of silica-cetuximab NPs (100 nm radius) in the presence of 10% FBS to breast cancer cell lines measured by flow cytometry.

Figure S16. Example of flow cytometry gating strategy in MDA-MB-468

Supplementary Methods

1. Scanning electron microscopy (SEM) imaging of sicastar®-greenF NPs.

SEM on commercially available sicastar®-greenF COOH-functionalized NPs was performed to determine the size dispersion of 50, 100 and 150 nm radius particles. Therefore, a small amount of stock NP solution was transferred to a small piece of silicon wafer attached to a SEM grid using conductive double-sided tape and dried at room temperature. The NPs were coated with a thin layer of gold (1 min sputter coating) and imaged using a FEI Quanta 3D FIB SEM/FIB microscope. The voltage was set at 5kV and a spot size of 4-4.5 was used in combination with a secondary electron detector.

2. Geometrical model of antibody accessibility

To better understand the accessibility of Fab sites to EGFR on a spherical NP, a simple geometrical model was constructed to simulate the number of accessible sites, n. The construction of the model is shown in Figure S1. First, we observe that for EGFRs to bind to Fabs, a region of space, shown in Figure S1A and B, must remain free. We name this region the exclusion region. We assume that this region is a sphere with a diameter equivalent to the distance between the furthest atoms in EGFR. If this region is occluded by the substrate (Figure S1B and D), the corresponding Fab site is deemed inaccessible. Figure S1C shows the geometrical model and the associated variables that parametrize it. In this way, we have reduced the EGFR-Cetuximab system to a geometrical problem that could in theory be applied to any Ig like antibody and any target.

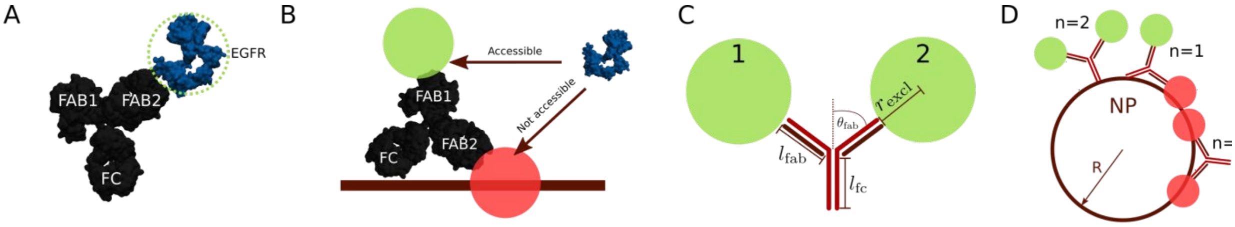

Figure S1. A) Molecular representation of EGFR bound to one of the Fab sites in Cetuximab. The dashed green circle shows the space that must remain free for an EGFR to be able to bind to the Fab site. B) When Cetuximab is deposited on a substrate, shown with a thick dark red line, for some orientations of the antibody, some Fab sites may be completely excluded from binding. C) The geometrical model assumes the antibody is three rigid rods, with regions that must remain free to be available for binding. The two binding sites are labelled 1 and 2. D) For some orientation of the antibody on a NP with radius R, one can count the number of accessible sites that remain unoccluded by the NP. Three states are possible: Two sites accessible ($n = 2$), one accessible ($n = 1$), and none accessible ($n = 0$).

The antibodies can orient along with three angles when they are deposited on the NP. The angles are shown in Figure S2. The values of the model used for the results shown in this paper are given in Table S1.

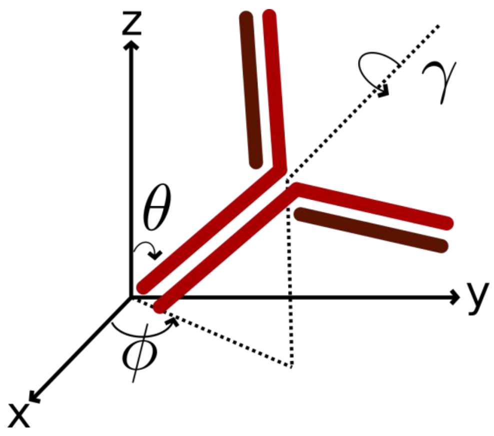

Figure S2. Three angles parametrize the orientation of the Cetuximab model. These angles are integrated over to measure the average accessibility number $\langle n \rangle$.

Parameter	Description	Value
R	NP radius	0-1000 nm
l_{fc}	FC stalk length	5 nm
l_{fab}	Fab stalk length	5 nm
θ_{fab}	Fab angle wrt vertical	$\frac{\pi}{3}$rad
r_{excl}	Exclusion zone radius	5 nm
θ	Azimuthal angle	$0-\pi$ rad
ϕ	Polar angle	$0-2\pi$ rad
γ	Axial rotation angle	$0-\pi$ rad

Table S1. Numerical values of the various parameters used in the model.

The number of accessible sites, n is then given by $n = a_1 + a_2$where a_i is the accessibility of Fab site i given by

$$a_i = \begin{cases} 1 & \text{if } d(\theta,\phi,\gamma) \geq r_{excl} + R \\ 0 & \text{otherwise.} \end{cases}$$

Above, $d(\theta,\phi,\gamma)$ is the distance between the center of the exclusion zone and the center of the NP. The NP is placed to touch the end of the antibody with the smallest z-coordinate value in the axes shown in Figure S2. When antibodies are deposited randomly on a substrate (spherical or otherwise), there is some chance that two antibodies land close enough to each other such that they block one or more of each other's binding sites. At this stage in the model, we assume that the density of antibodies on the surfaces of NPs is low enough to make this probability negligible. We can check the assumption with the following simple calculation:

Assume that upon deposition, each antibody occupies a disk of radius 5 nm. Our smallest NPs are on average 50 nm in radius, making their surface area approximately 31415 nm^2. For spherical surfaces, the maximum density packing is roughly 83% (random close packing on a sphere), meaning that the maximum number of antibodies (lying flat on the NP) without any steric hindrance is roughly 332. This number is the approximate *theoretical maximum* number of antibodies we can deposit on a NP without any steric hindrance or overlap of exclusion zones between antibodies. Our experimental dSTORM measurements place the total number of antibodies for NPs of radius 50 nm at approximately 194 (Table S4). This calculation is repeated for all values of NP radius that we analyzed in Table S2. Note that we are always far from full coverage. Given the results in Table S2, we assume that we can ignore the effect of overlap between antibodies on NP surfaces for our purposes.

Avg. NP Radius (nm)	Average number of ABs/NP	Theoretical Maximum
50	194	332
100	215	1328
150	792	2988

Table S2: Measured and theoretical maximum number of antibodies per NP as a function of NP radius.

Since we assume that overlap between antibodies is negligible, the problem of estimating averaged over all antibodies can be reduced to the average accessibility number of a *single* antibody, averaged over all three orientations θ, ϕ, and γ. We calculated the average number as

$$\langle n \rangle = \frac{1}{4\pi^2}\int_0^{\pi} d\theta \int_0^{2\pi} d\phi \int_0^{\pi} d\gamma \sin\theta\, n(\theta,\phi,\gamma)\; p(\theta,\phi,\gamma).$$

We further assume that there is no preference in the orientation of the antibody on NP surfaces, *i.e.* that $p(\theta,\phi,\gamma) = 1$. The above equation is integrated with Monte Carlo integration for the parameters given in Table S1. The results of this calculation are shown in Figure S3A. We see that the expected number of accessible Fab sites for the NP radii we used to lie between 0.9 and 1 per antibody, corresponding to 45% to 50% accessibility. The closeness of these measurements to experimental values suggests that such a model can be used to augment future experimental studies. While this accessibility may be adequate for several applications, we can use this model to understand how this system can be changed to improve the number of

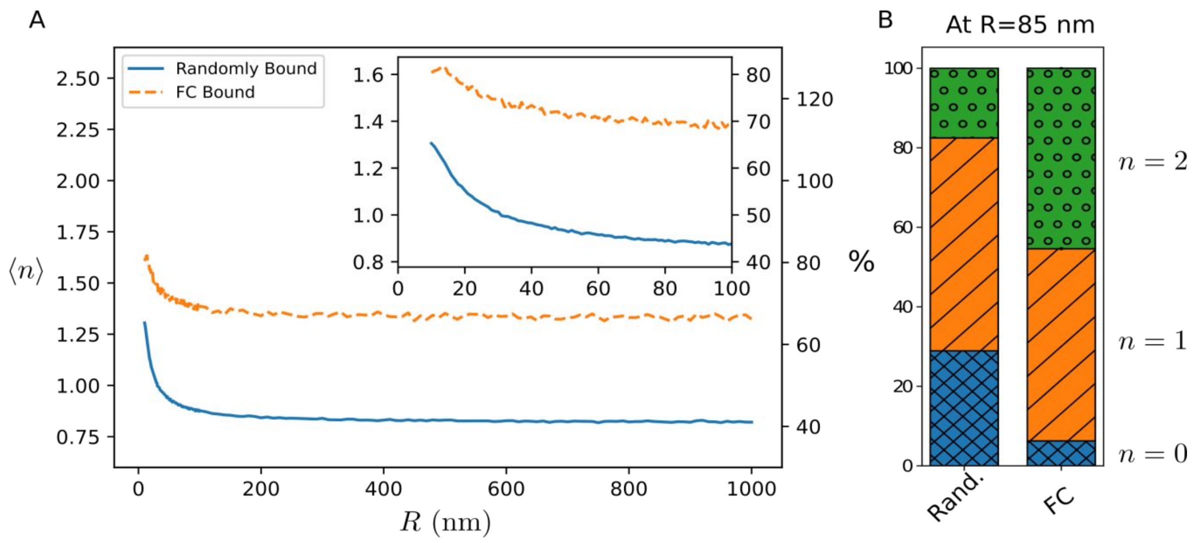

Figure S3. Estimates of the number of accessible Fab sites. A) Average number of accessible Fab sites as a function of NP radius R. Inset shows the results for R<100 nm. If Cetuximabs are deposited randomly with random orientations, we obtain the blue line which shows an accessibility of 45% to 50% for our NPs. This can be improved almost two-fold by forcing binding only at the FC end. B) shows that forcing binding only at the FC end almost eliminates non-accessible ($n = 0$*) states, as a percentage, and replaces them with* $n = 2$ *states. n= number of accessible Fab sites.*

accessible Fab sites (or antibody accessibility in general). A simple adjustment could be to constrain the binding such that it can only occur at the FC end (similar to what is shown in Figure S1C). Can we eliminate $n = 0$ states in this manner? While challenging to enforce experimentally, we can use the geometrical model to study this change. The binding to the FC end can be enforced by returning to the assumption $p(\theta,\phi,\gamma) = 1$. We can change this probability distribution to represent no Fab end binding between antibody and NP as follows. Let

$$p(\theta,\phi,\gamma) = \frac{1}{V}\begin{cases} 1 & \text{if} \min\left(z_{fab1}, z_{fab2}, z_{fc}\right) = z_{fc} \\ 0 & \text{otherwise} \end{cases},$$

where z_{fab1}, z_{fab2}, and z_{fc} are the lowest z coordinates of the two Fab domains and the FC stalk, respectively and

$$V = \frac{1}{4\pi^2}\int_0^{\pi} d\theta \int_0^{2\pi} d\phi \int_0^{\pi} d\gamma \sin\theta\, p(\theta,\phi,\gamma),$$

is a normalization factor. This new probability distribution is then plugged into the equation for $\langle n \rangle$ to estimate the improvement in accessibility. The results in Figure S3A show that we can expect a two-fold improvement in accessibility. One can drill deeper to see the breakdown of the percentage of $n = 2, n = 1$, and $n = 0$ states to understand where this improvement in accessibility comes from. Figure S3B shows that by eliminating binding to Fab ends, we almost eliminate $n = 0$ states.

3. Synthesis of PLGA NPs

PLGA NPs were synthesized *via* nanoprecipitation as previously described.[1–3] Briefly, polymers were dissolved in acetonitrile at a concentration of 10 mg/ml. Polymers were mixed at a ratio of 15% PLGA, 30% PLGA-PEG-COOH and 55% PLGA-PEG (1K PEG length). To identify the position of PLGA-NPs, DiO was added to the polymer mixture at a final concentration of 10 µM. Next, 300 µl of polymer-DiO mixture was added to 3 ml of MilliQ water while stirred at 400 rpm using a magnetic stirring bar (final polymer concentration 1 mg/ml). The acetonitrile was extracted by solvent evaporation overnight at room temperature under continuous stirring. PLGA NPs were purified by centrifugation at 16000g and 19°C for 12 minutes to remove non-encapsulated DiO dye and stored in MilliQ at 4C until further use. PLGA-NPs size was measured with a Malvern Zetasizer Nano ZS (Malvern Instruments) in a ZEN2112 cuvette. The measurement was performed in triplicate, with 10 runs for each measurement.

4. Conjugation of antibodies to PLGA NPs

Antibodies (Cetuximab-AF647, Cetuximab plain or anti-mouse control antibody) were conjugated *via* EDC coupling chemistry as described for silica-COOH NPs. Briefly, 200 µg PLGA NPs were centrifuged a 16000g and 19°C for 12 minutes and resuspended in MES buffer (50 mM, pH 5) containing 2 mM EDC and incubated for 15 minutes at 22°C and 400 rpm in a ThermoMixer®. After the EDC activation, NPS were sonicated for 5 minutes in a

bath sonicator. 40 µg Cetuximab-AF647, Cetuximab plain or anti-mouse control antibody were added to EDC-activated PLGA NPs and incubated for 2h at 22°C and 400 rpm in a ThermoMixer®. Unconjugated antibody was purified by washing with 25 mM HEPES buffer and centrifuging thrice at 16000g for 12 minutes. PLGA NPs were resuspended at a final concentration of 1 mg/ml in 25 mM HEPES buffer and stored at 4°C until further use.

5. Incubation of PLGA NPs with EGFR-AF647 probe.

The functionality of cetuximab antibodies conjugated to PLGA NPs was studied as previously described for silica NPs. 10 µl PLGA NPs (1 mg/ml) functionalized with cetuximab plain or anti-mouse control antibody were incubated with 20 pmol EGFR probe and 0.5% bovine serum albumin for 1h at 15°C and 400 rpm in a ThermoMixer®. NPs were sonicated in a bath sonicator for 5 minutes to aid redispersion and imaged the same day.

6. TIRF and dSTORM imaging of PLGA NPs

Imaging chambers for TIRF imaging were prepared as previously described for dSTORM imaging of NPs in the main text. Additionally, imaging chambers were passivated with 100% FBS for 5 minutes to minimize background fluorescence coming from free proteins in solution, followed by washing with 100 µl HEPES 25 mM buffer. PLGA NPs conjugated with antibodies were added to the imaging chamber (1:10 dilution from stock) and allowed to adsorb for 20 minutes at room temperature. The imaging chamber was then rinsed with 200 µl HEPES 25 mM to remove non attached NPs. TIRF images of the 488 (DiO) and 647 (AF647) channels were acquired at 2% laser power and 100 ms exposure. For PLGA NPs conjugated with plain cetuximab and anti-mouse control antibody incubated with EGFR-AF647, dSTORM imaging was performed as previously described for silica NPs. First, imaging chambers were rinsed with STORM buffer (50 mM Tris pH 8, 10 mM NaCl, 10% w/v glucose, 50 mM cysteamine, 0.5 mg/ml glucose oxidase, 40 µg/ml catalase). Flow chambers were then sealed with nail polish to prevent solvent evaporation. dSTORM images were acquired for 30000 frames at 30 ms exposure and 100% laser power for the 647 channel. The fluorescent PLGA NPs were used to identify the NP position and drift correction, and were imaged one frame every 100 frames with the 488 laser at 5% laser power.

7. dSTORM analysis of PLGA NPs

dSTORM analysis was performed as previously described in the main text for silica NPs with a slight adjustment. For NP clustering, the Bandwidth was adjusted to 100 nm and clusters with less than 10 localizations were discarded. Next, the number of 647 localizations were counted around each NP center at a maximum radius of 200 nm. The data was plotted in histograms using Origin 2020 and fitted with an exponential decay function.

Supplementary Figures

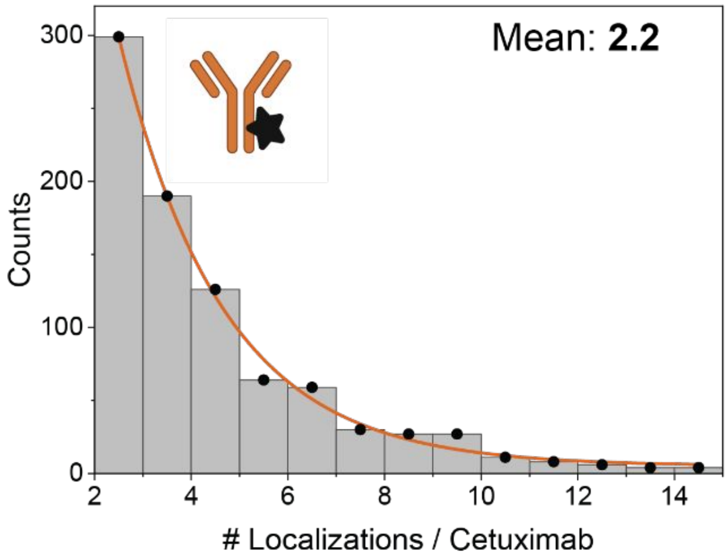

Figure S4. Quantification of dSTORM localizations per single cetuximab antibody. The histogram of the number of localizations per cetuximab was fitted with an exponential decay function to obtain the mean.

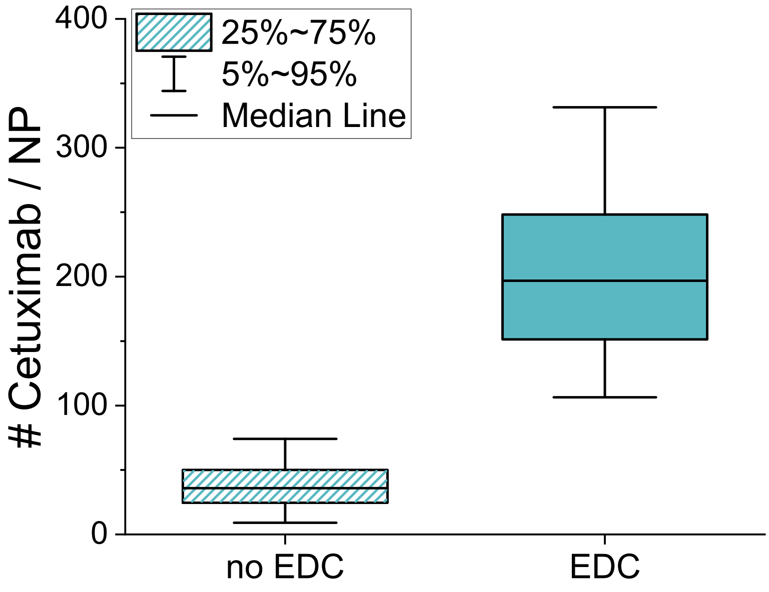

Figure S5. dSTORM quantification of cetuximab-AF647 binding to 100 nm radius silica NPs without and with the covalent coupling agent 1-ethyl-3-(3-dimethylaminopropyl)-carbodiimide (EDC). The concentration of cetuximab added during the conjugation was 3437 cetuximab/ NP. Numbers of cetuximabs were measured at the single-particle level.

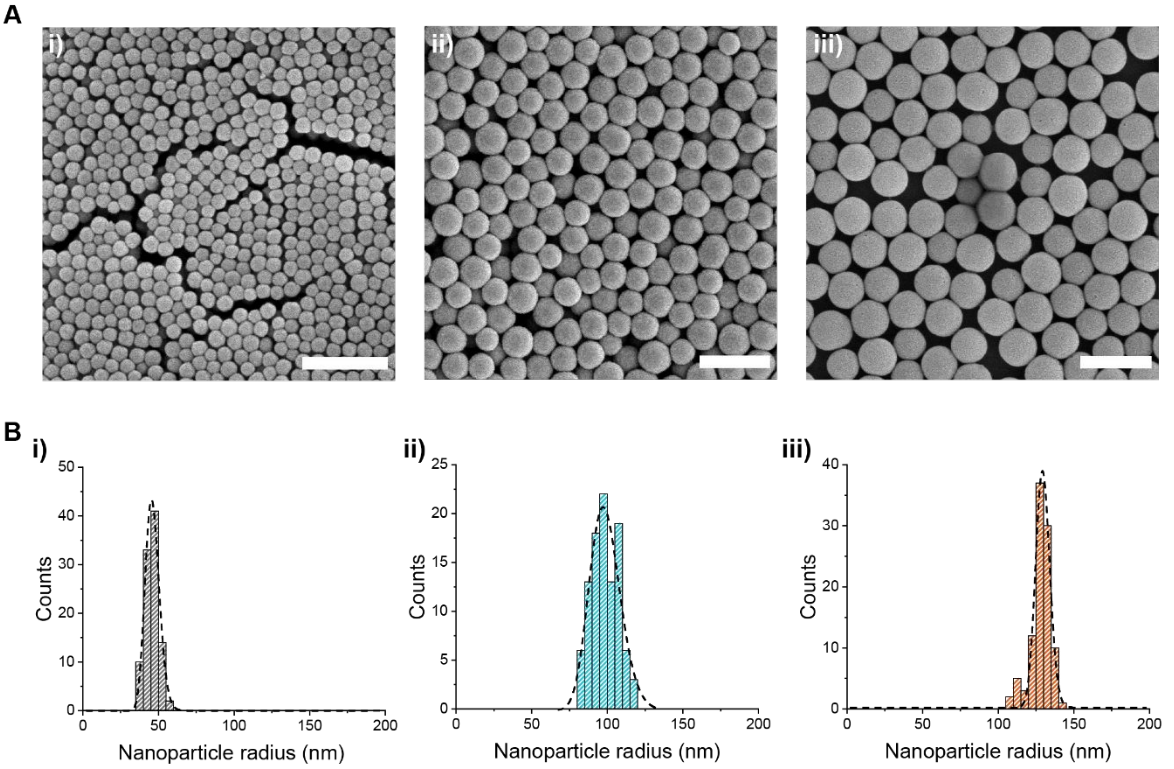

Figure S6. Size dispersion of silica-COOH NPs measured by SEM. A) Representative SEM images of 50 (i), 100 (ii) and 150 (iii) nm radius silica particles at 12000X, 10000X and 10000X magnification, respectively. Scale bar 500 nm. B) Histograms of the NP radius of 50 (i), 100 (ii) and 150 (iii) nm radius silica particles. Distributions were fitted with a Log normal function to extract the mean.

Sample	N	Fitting	R-square	Mean radius (nm)	SD	% Coefficient of Variation (CV)
50 nm radius	100	Log Normal	0.99	46.1	4.7	10.2
100 nm radius	100	Log Normal	0.94	98.8	10.2	10.3
150 nm radius	100	Log Normal	0.99	129.5	4.7	3.6

Table S3. Coefficient of variation (CV, %) of the NP radius measured by SEM for NPs with different radii. N represents the number of analyzed NPs. Histograms depicted in figure S2 were fitted using a Log normal distribution to extract the mean, standard deviation (SD) and R-square values.

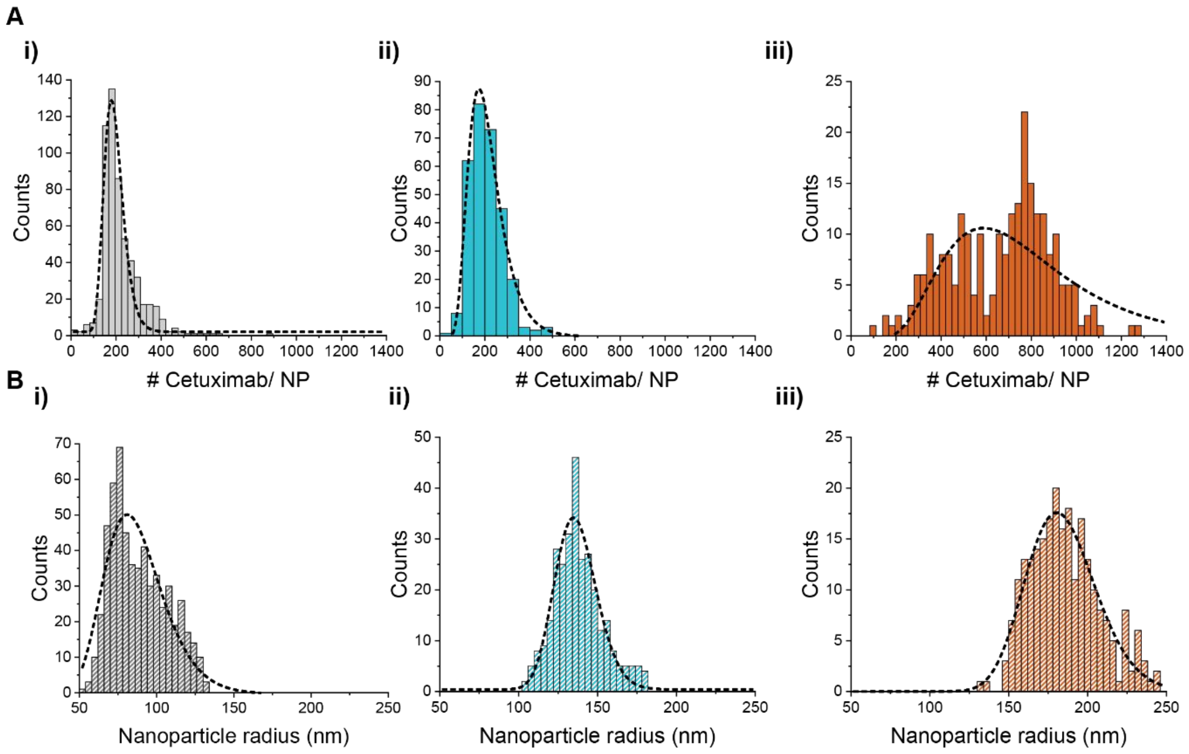

Figure S7. Number of cetuximab per NP and NP size measured by dSTORM. A) Histograms of the number of cetuximab per NP of 50 (i), 100 (ii) and 150 (iii) nm radius silica particles. B) Histograms of the NP radius after cetuximab conjugation of 50 (i), 100 (ii) and 150 (iii) nm radius silica particles. Distributions were fitted with a Log normal function to extract the mean.

Sample	Measurement	N	Fitting	R-square	Mean	SD	% Coefficient of Variation (CV)
50 nm radius	Cetuximab/ NP	574	Log Normal	0,95	193.7	44.4	22.9
	NP radius (nm)	574	Log Normal	0,89	87.5	20.1	22.9
100 nm radius	Cetuximab/ NP	299	Log Normal	0,99	214.7	83.9	39.1
	NP radius (nm)	299	Log Normal	0,94	136.7	13.3	9.7
150 nm radius	Cetuximab/ NP	247	Log Normal	0,53	791.6	370.3	46.8
	NP radius (nm)	247	Log Normal	0,94	184.5	22.5	12.2

Table S4. Coefficient of variation (CV, %) of the number of cetuximab and NP radius measured by dSTORM for NPs with different radii. N represents the number of analyzed NPs. Histograms depicted in figure S3 were fitted using a Log normal distribution to extract the mean, standard deviation (SD) and R-square values.

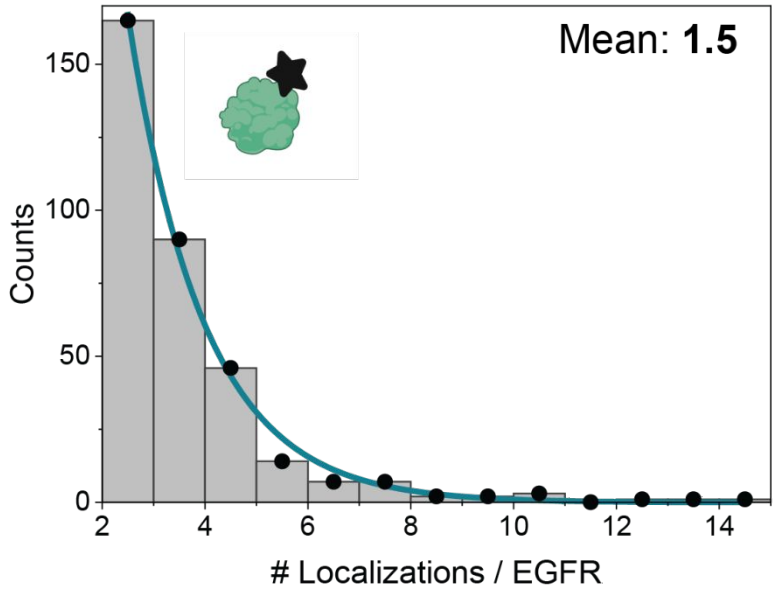

Figure S8. Quantification of dSTORM localizations per single recombinant EGFR probe. The histogram of the number of localizations per EGFR was fitted with an exponential decay function to obtain the mean.

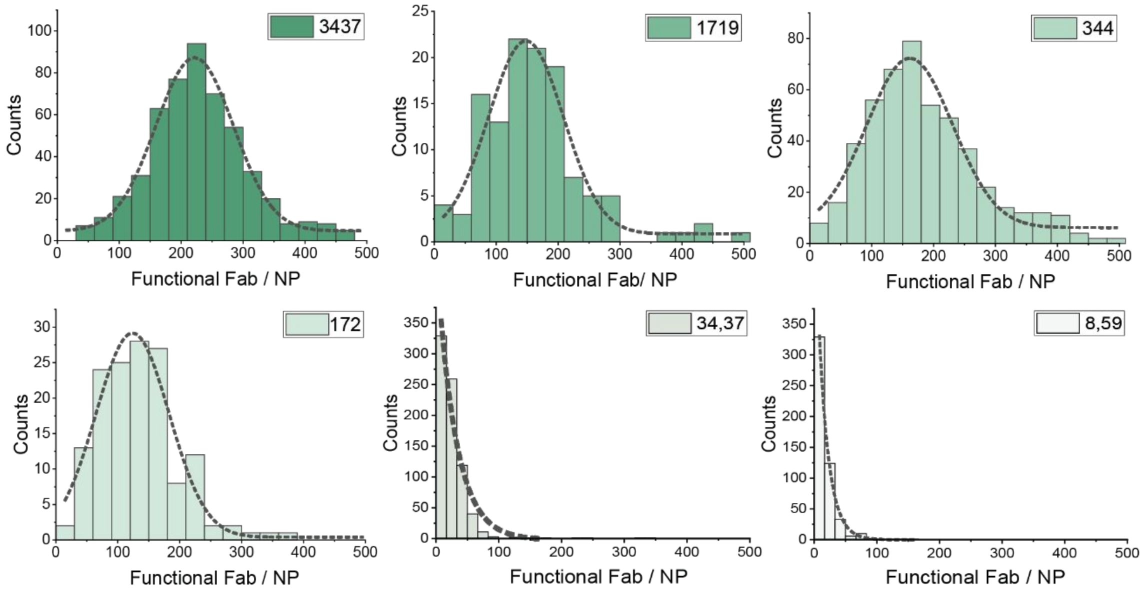

Figure S9. Distributions of functional Fab fragments at different added cetuximab concentrations in the conjugation reaction. The number in the legend represents the number of cetuximab added per NP. Distributions were fitted with a gaussian (sample 3437, 1718, 344 cetuximab/ NP added) or exponential decay fitting (sample 34,37 and 8,59 cetuximab / NP added).

Sample (cetuximab/ nanoparticle)	N	Fitting	R-square	Mean	SD
3437	519	Gaussian	0.98	221.7	125.6
1719	121	Gaussian	0.92	148.1	121.0
344	491	Gaussian	0.96	161.5	138.8
172	146	Gaussian	0.94	123.9	118.0
34,37	769	Exponential decay	0.97	30.3	30.3
8,59	508	Exponential Decay	0.99	16.0	16.0

Table S5. Mean and SD of functional Fab fragments extracted from histogram distribution fittings. N represents the number of analyzed NPs. Histograms depicted in figure S5 were fitted using a Gaussian or exponential decay distribution.

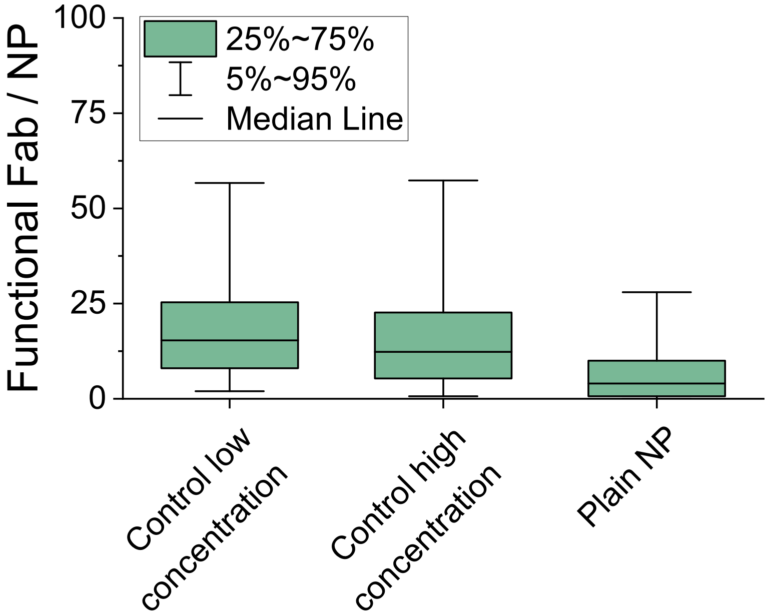

Figure S10. Binding of EGFR probe to control NPs and silica-COOH NP without antibody (Plain NP). Control low and high concentration NPs consist of 34,37 and 3427 anti-mouse antibodies / NP added to the conjugation reaction, respectively.

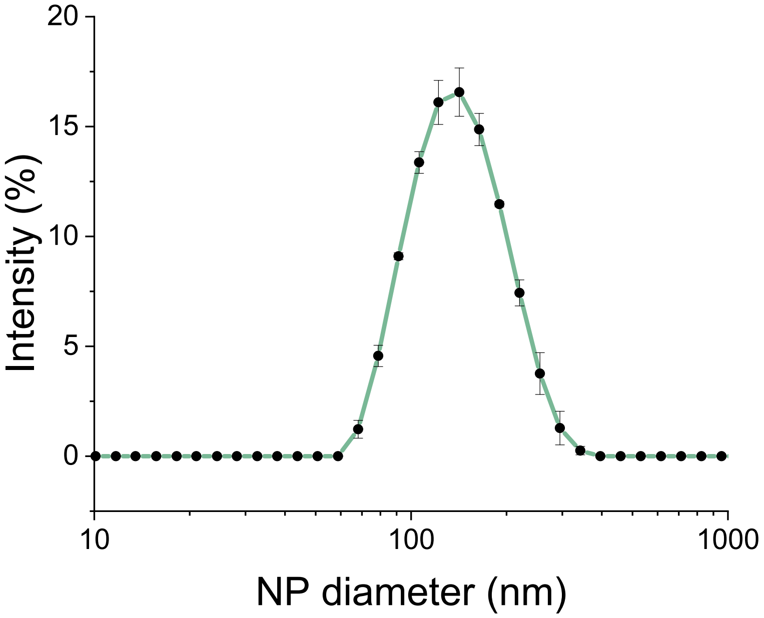

Figure S11. Hydrodynamic diameter of formulated PLGA-COOH NPs measured by dynamic light scattering. X-axis is shown in log scale. The average of 3 measurements yielded a mean NP diameter of 132,7 nm (SD 0.66 nm). The measured PDI was 0.094 (SD 0.027).

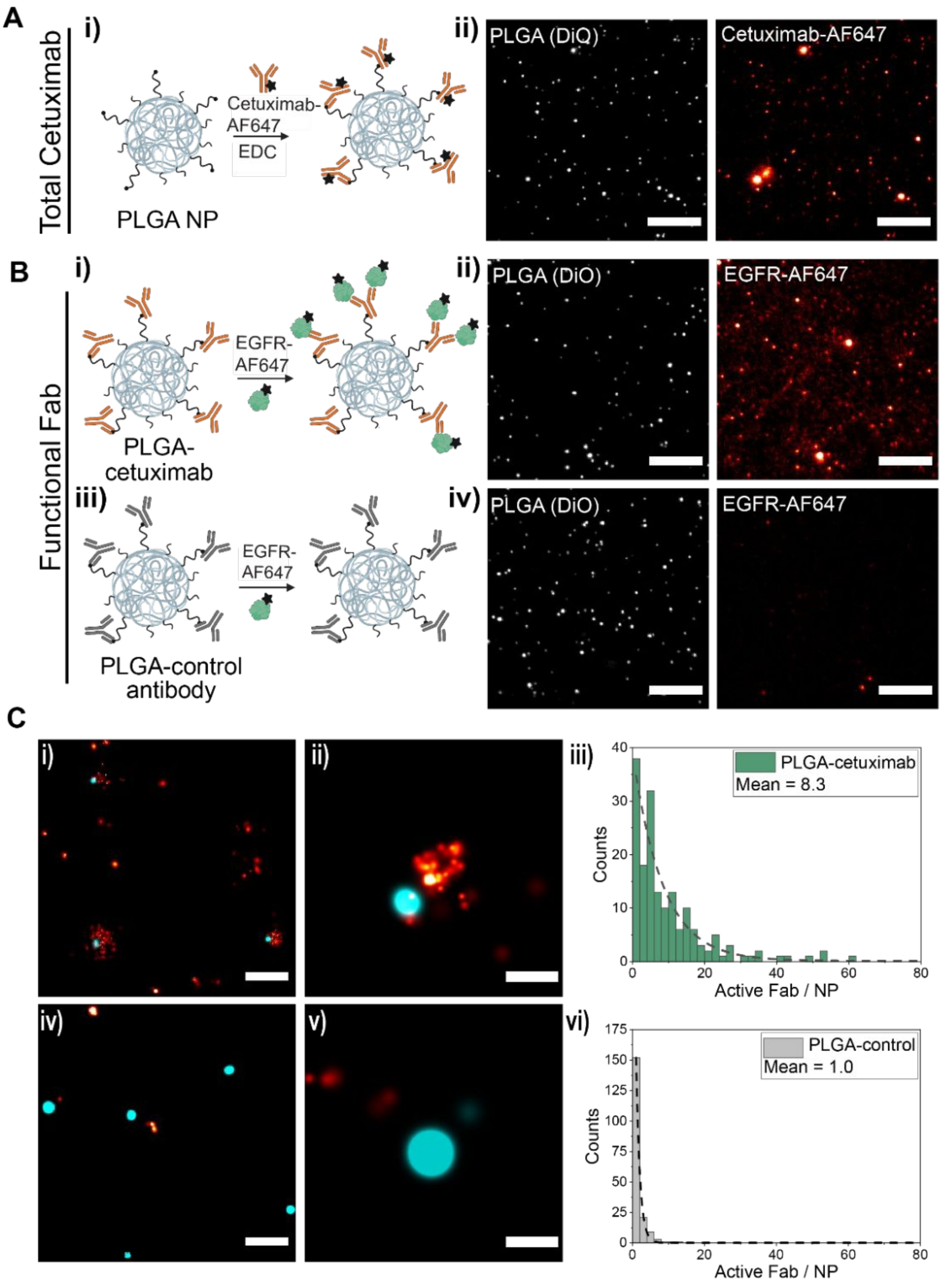

Figure S12. dSTORM imaging of cetuximab-conjugated PLGA NPs. A) TIRFimaging of total cetuximab-AF647-conjugated PLGA NPs. i) Schematic representation of cetuximab-AF647 conjugation to PLGA NPs mediated by EDC coupling chemistry. ii) TIRF imaging of PLGA NPs (DiO signal) and cetuximab-647 antibodies conjugated to PLGA NPs. The signal originating from cetuximab-647 antibodies correlates to the PLGA NP position, indicating successful antibody conjugation. B) TIRF imaging of EGFR functionality assay of cetuximab (i-ii) and control (iii-iv) antibody-functionalized PLGA NPs. i) Schematic representation of NP functionality assay of cetuximab-functionalized PLGA NPs. ii) TIRF imaging of PLGA NPs (DiO signal) and EGFR-647 probe binding to cetuximab-functionalized PLGA NPs. High-intensity EGFR-647 spots correlate with the PLGA NP position, while some background fluorescence is detected on the glass surface. iii) Schematic representation of NP functionality assay of control antibody-functionalized PLGA NPs. iv) TIRF imaging of PLGA NPs (DiO signal) and EGFR-647 probe binding to control antibody-functionalized PLGA NPs. EGFR-647 signal does not correlate with PLGA NP position, indicating background fluorescence on the glass. C) dSTORM imaging and quantification of EGFR functionality assay of cetuximab (i-iii) and control (iv-vi) antibody-conjugated PLGA NP. PLGA-NP position is illustrated in Cyan for reference and EGFR-localizations in red. Scale bar 500 nm (i and iv) and 100 nm (ii and v). Distributions of active Fab fragments for cetuximab (iii, N= 174 NPs) and control antibody (vi, N=190 NPs). Distributions were fitted with an exponential decay fitting, revealing a mean number of functional Fab fragments of 8.3 (R-square 0.91) and 1.0 (R-square 0.99), respectively.

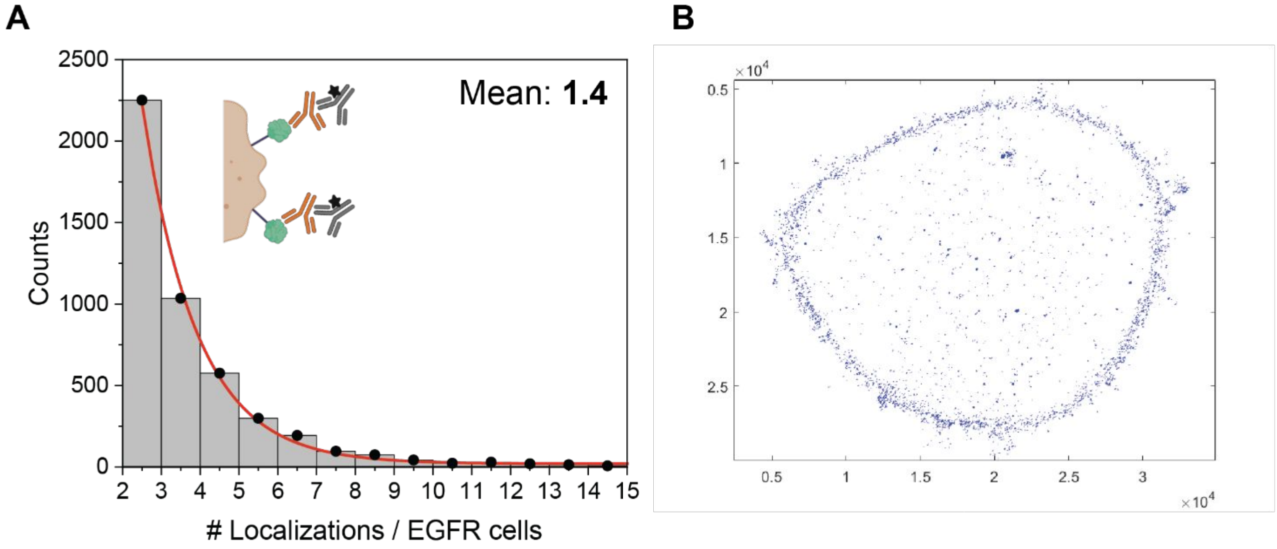

Figure S13. Number of localizations / EGFR measured in low concentration cetuximab staining of MDA-MB-468 cells. A) Histogram representing number of localizations / EGFR. The histogram was fitted with an exponential decay function to obtain the mean. In total, 6 cells were measured. B) Representative image of MDA-MB-468 cell used for EGFR calibration. X and Y-axis represent spatial coordinates (nm) of the detected dSTORM localizations. MDA-MB-468 cells were stained with 0.01 µg/ml cetuximab and 1:150 diluted Alexa Fluor 647 secondary anti-mouse antibody. On average, 20,7 localizations / µm² were obtained at this low concentration cetuximab labeling.

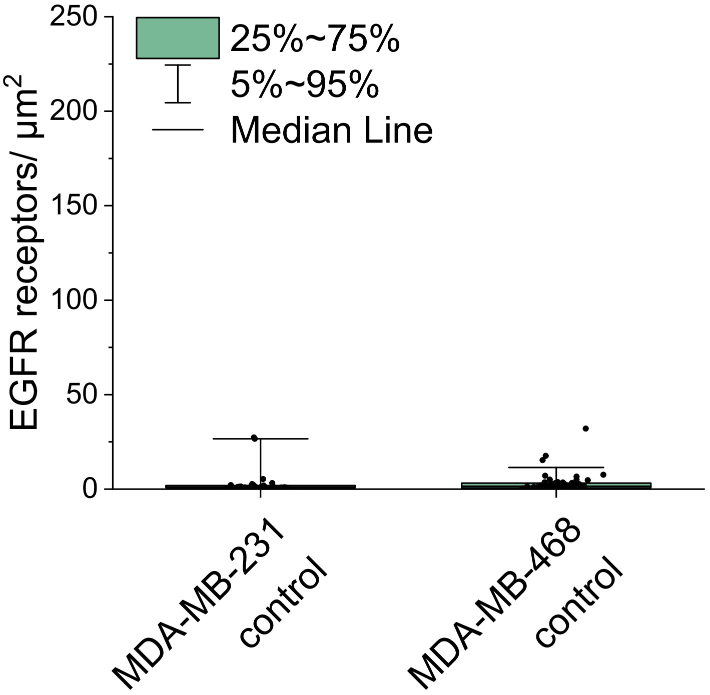

Figure S14. Unspecific binding control of Alexa647-labeled secondary antibody to MDA-MB-231 and MDA-MB-468 cells.

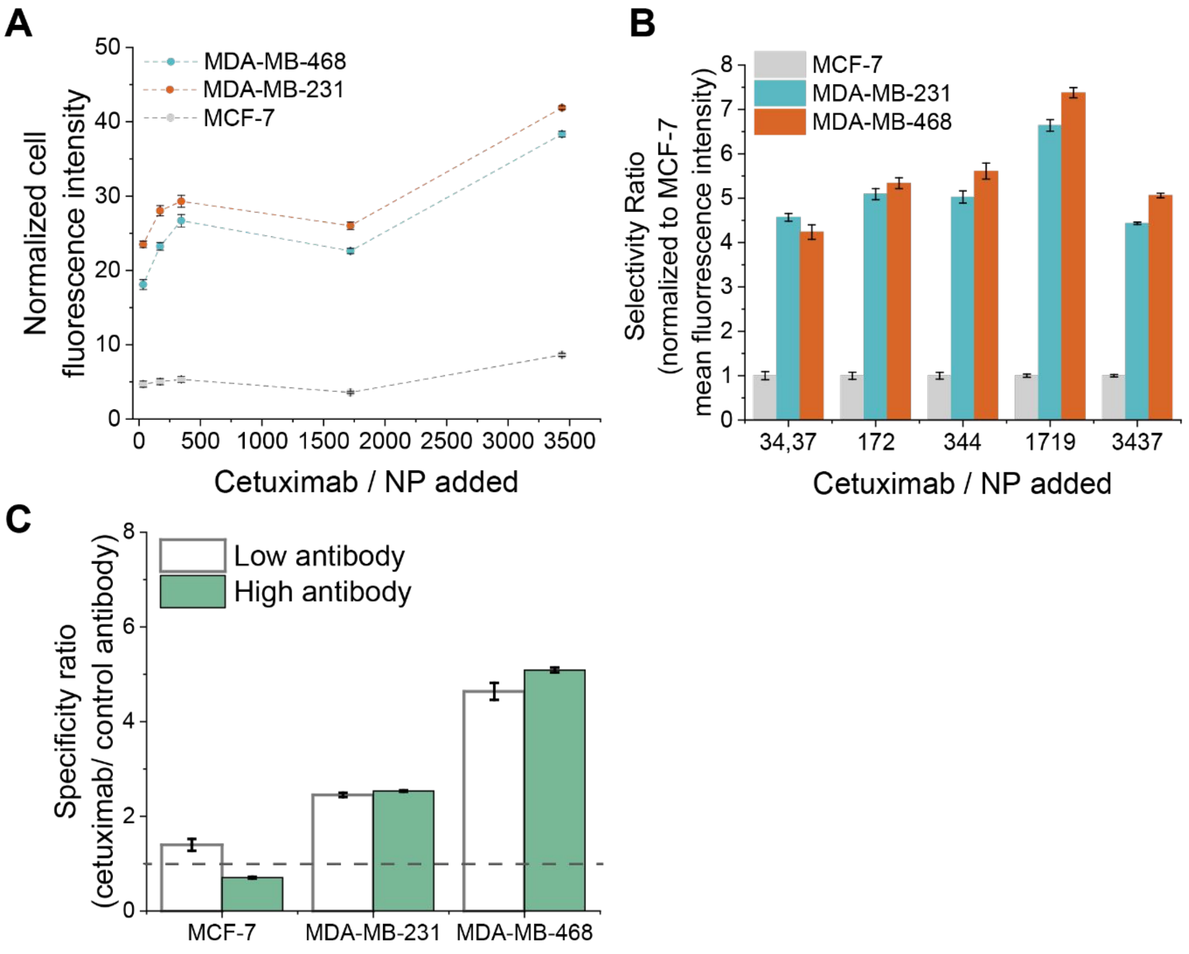

Figure S15. Targeting of silica-cetuximab NPs (100 nm radius) in the presence of 10% FBS to breast cancer cell lines measured by flow cytometry. A) Normalized cell fluorescence intensity after 90 min of silica-cetuximab incubation at different cetuximab conjugation amounts. Mean fluorescence intensity was normalized with respect to cells without NPs. B) Selectivity ratio of silica-cetuximab uptake normalized to MCF-7 cells. C) Specificity ratio of silica-cetuximab at low (34,37 antibodies/NP) and high (3427 antibodies/NP) antibody conjugation compared to control antibody conjugated NPs. A line was added for visualization purposes at specificity = 1 (no difference compared to control antibody NPs).

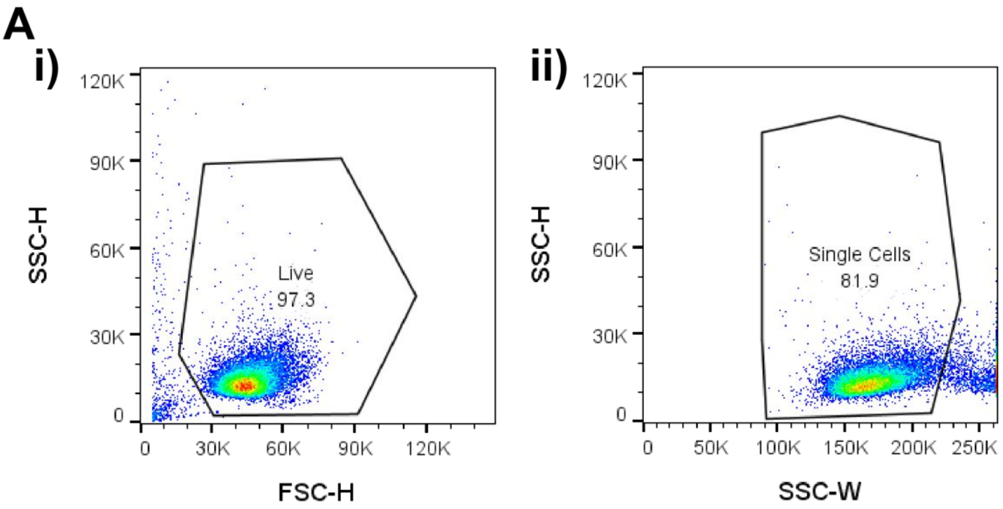

Figure S16. Example of flow cytometry gating strategy in MDA-MB-468 (i) Debris was excluded from the side versus forward scatter plot (SSC-H versus FSC-H) (ii) cell clusters were excluded from the side scatter height versus side scatter width plot (SSC-H versus SSC-W). Data shown are representative of cells with no NPs added.

Supplementary References

(1) Andrian, T.; Delcanale, P.; Pujals, S.; Albertazzi, L. Correlating Super-Resolution Microscopy and Transmission Electron Microscopy Reveals Multiparametric Heterogeneity in Nanoparticles. *Nano Lett.* **2021**, acs.nanolett.1c01666. https://doi.org/10.1021/acs.nanolett.1c01666.

(2) Andrian, T.; Pujals, S.; Albertazzi, L. Quantifying the Effect of PEG Architecture on Nanoparticle Ligand Availability Using DNA-PAINT. *Nanoscale Adv.* **2021**, 10.1039.D1NA00696G. https://doi.org/10.1039/D1NA00696G.

(3) Glinkowska Mares, A.; Pacassoni, G.; Marti, J. S.; Pujals, S.; Albertazzi, L. Formulation of Tunable Size PLGA-PEG Nanoparticles for Drug Delivery Using Microfluidic Technology. *PLOS ONE* **2021**, *16* (6), e0251821. https://doi.org/10.1371/journal.pone.0251821.
